# MT-HESS: an efficient Bayesian approach for simultaneous association detection in OMICS datasets, with application to eQTL mapping in multiple tissues

**DOI:** 10.1093/bioinformatics/btv568

**Published:** 2015-10-26

**Authors:** Alex Lewin, Habib Saadi, James E. Peters, Aida Moreno-Moral, James C. Lee, Kenneth G. C. Smith, Enrico Petretto, Leonardo Bottolo, Sylvia Richardson

**Affiliations:** ^1^Department of Mathematics, Brunel University London,; ^2^Department of Epidemiology and Biostatistics, Imperial College London, London,; ^3^Cambridge Institute for Medical Research, University of Cambridge, Cambridge,; ^4^MRC Biostatistics Unit, Cambridge Institute of Public Health, Cambridge,; ^5^MRC Clinical Sciences Centre, Imperial College London, London, UK,; ^6^Duke-NUS Graduate Medical School, Singapore, Singapore,; ^7^Department of Mathematics, Imperial College London, London, UK and; ^8^Department of Medical Genetics, University of Cambridge

## Abstract

**Motivation:** Analysing the joint association between a large set of responses and predictors is a fundamental statistical task in integrative genomics, exemplified by numerous expression Quantitative Trait Loci (eQTL) studies. Of particular interest are the so-called **‘**hotspots**’**, important genetic variants that regulate the expression of many genes. Recently, attention has focussed on whether eQTLs are common to several tissues, cell-types or, more generally, conditions or whether they are specific to a particular condition.

**Results:** We have implemented MT-HESS, a Bayesian hierarchical model that analyses the association between a large set of predictors, e.g. SNPs, and many responses, e.g. gene expression, in multiple tissues, cells or conditions. Our Bayesian sparse regression algorithm goes beyond **‘**one-at-a-time**’** association tests between SNPs and responses and uses a fully multivariate model search across all linear combinations of SNPs, coupled with a model of the correlation between condition/tissue-specific responses. In addition, we use a hierarchical structure to leverage shared information across different genes, thus improving the detection of hotspots. We show the increase of power resulting from our new approach in an extensive simulation study. Our analysis of two case studies highlights new hotspots that would remain undetected by standard approaches and shows how greater prediction power can be achieved when several tissues are jointly considered.

**Availability and implementation:** C++ source code and documentation including compilation instructions are available under GNU licence at http://www.mrc-bsu.cam.ac.uk/software/**.**

**Contact:** sylvia.richardson@mrc-bsu.cam.ac.uk or lb664@cam.ac.uk

**Supplementary information:** Supplementary data are available at *Bioinformatics* online.

## 1 Introduction

Integrating different layers of genomic information is essential to improve our understanding of the genetic basis of complex diseases. The development of integrative analysis strategies has become an important part of experimental design in the era of next-generation genomics (Hawkins *et* *al.*, 2010). A fundamental task faced by many integrative strategies in genomics is to study the associations between two high-dimensional datasets. The study of such associations and the related biological questions can be naturally built within a regression framework, which exploits prior biological knowledge on the direction of the relationships between the different layers of genomics data.

One prime example is expression quantitative trait loci (eQTL) analysis, which links DNA polymorphisms to gene expression levels on a genome-wide basis. Typically, such analyses would involve testing for association of all transcript-SNP pairs. When eQTL studies involve large numbers of variants and genes, performing millions of such tests can become slow and it has been necessary to develop fast implementations (Shabalin, 2012). To go beyond such ‘one at-a-time’ strategies, eQTL mapping can be performed using high-dimensional regression models with expression measurements modelled as responses and genetic variants as predictors (Bottolo *et* *al.*, 2011; Fusi *et al.*, 2012; Scott-Boyer *et* *al.*, 2012). The high-dimensional regression framework leverages information across the set of responses that are related to the same predictor, increasing the probability of detecting hotspots, i.e. genomic loci that regulate several genes at once.

There is currently much interest in dissecting tissue, cell type or condition specificity of eQTLs (Fairfax *et* *al.*, 2012; Lee *et* *al.*, 2014; Melé *et* *al.*, 2015; Raj *et al.*, 2014; Westra *et* *al.*, 2013). The multiple tissues or cell types add an extra dimension to the data. For instance, analysis of eQTLs across multiple tissues or cell types can inform to which extent eQTLs are shared (or conserved) between different systems, therefore elucidating fundamental genetic regulatory mechanisms. So far these questions have been addressed using one of two alternative approaches. The first uses separate analysis for each tissue, and intersects the resulting lists of genetic variants (Fairfax *et* *al.*, 2012). The second approach (Flutre *et* *al.*, 2013; Petretto *et* *al.*, 2010) combines tissues but performs a separate analysis for each predictor (genetic variant) and response (gene expression level). Neither approach fully exploits the data available, nor both can result in a loss of power to detect small effect eQTLs that are shared across conditions. To the best of our knowledge, there is currently no alternative method that goes beyond ‘one-at-a-time’ association tests and models *simultaneously* the expression of multiple genes under multiple conditions in a multivariate way.

In this article, we propose a generic Bayesian variable selection approach and an associated evolutionary stochastic search algorithm to tackle the challenging integrative task of linking parallel high-dimensional multivariate regressions in a computationally efficient way. The specificity of our approach is: (i) to move away from single feature at-a-time analysis and account for the correlated nature of the predictors by implementing a fully multivariate model search over the space of predictors (ii) to allow the analysis of multi-dimensional responses, and (iii) to exploit the *relatedness* of multiple responses through a Bayesian hierarchical model. Hierarchical modelling of expression responses allows us to exploit the potential functional relationships (e.g. co-regulation relationship, mRNA–mRNA interactions, protein–protein interactions, etc.) between multiple genes, thus increasing the power to detect hotspots. We build on our previous work (Bottolo *et al.*, 2011; Petretto *et* *al.*, 2010), demonstrate the power of our new efficient hierarchical implementation in an extensive simulation study when compared with MANOVA or the intersection of condition/tissue-specific results. We also illustrate the benefits of our approach in two case studies related to eQTLs in multiple conditions (tissues or cell types), where we recapitulate previously validated hotspots and uncover new hotspots. In one case study, we also show that when several tissues are jointly considered the prediction error is substantially reduced compared with separate single-tissue analysis.

Even though our case studies relate to eQTL analyses, the model and algorithm that we present are generic and can be used for a large range of integrative genomics analyses that can be formulated within a parallel regression framework, such as finding targets for miRNAs by regressing gene expression levels on miRNA levels (Stingo *et* *al.*, 2010), linking DNA variation and metabolites levels, the so-called mQTL analyses (Marttinen *et* *al.*, 2014), or linking copy number alterations and tumour gene expression (Kristensen *et* *al.*, 2014).

## 2 Modelling approach

We describe here in a non-technical fashion how we model the association between multiple responses, here the expression of genes in different conditions (e.g. tissues, cell-types, disease states, etc.), and many predictors, here the SNPs. We present a Bayesian variable selection method that acts simultaneously on three levels:

1. Each response in each tissue is sparse-regressed on all the predictors.
2. The sparse regressions from all tissues for the same response are performed *jointly* as a multi-variate regression, modelling the correlation between tissues, with the same predictors selected for controlling the response in all tissues.
3. The multi-tissue regressions across all responses are influenced by shared prior parameters that encourage borrowing of information.

Combining information between tissues allows us to boost signal in a robust way because the residual correlation is modelled accurately by latent covariance matrices.

The first level is the key driver of the method. This single response variable selection is accurately described in Bottolo and Richardson (2010). Building on a sparse formulation, this level of analysis eliminates all predictors for which the signal is not strong enough. The model takes into account the correlation between predictors to better identify the best supported combination of predictors. The performance of this method is illustrated in Bottolo and Richardson (2010) and Bottolo *et* *al.* (2013), and it shows a major improvement over univariate and commonly used penalized regression methods used in the ‘large *p*, small *n*’ framework in terms of separation between signal and background noise, and in terms of genetic resolution as it can handle the highly correlated predictors that result from linkage disequilibrium (LD).

The second level is built on top of this variable selection, in the sense that evidence from all the tissues influences the probability for a predictor to be selected. This strong assumption is reasonable only when considering a small number of correlated traits for which evidence of *joint control* is interesting to quantify. Note that once a predictor is selected, tissue-specific regression coefficients can be estimated from the posterior distribution.

The third level pools information across all responses in order to enhance the detection of hotspots. It also has the great benefit of eliminating many false positives. The performance of this choice of prior was explored in Bottolo *et* *al.* (2011).

## 3 Methods

### 3.1 Bayesian hierarchical sparse regressions

Our model is an extension of HESS algorithm (Bottolo *et al.*, 2011; Richardson *et* *al.*, 2010) to the case where the response variables are observed in multiple conditions, e.g. in different tissues, cell types or time points.

We consider *q* response variables observed in *r* different conditions. In the following, we will use upper-case letters for matrices and lower-case letters for vectors and scalars. For k=1,…,q, let *Y_k_* be an n×r matrix, whose entry $y_{ik\ell }$ is the response *k* measured in condition ℓ for individual *i.* The explanatory variables are stored in an *n* × *p* matrix *X* such that $x_{ij}$ is the *j*th explanatory variable measured in individual *i.* The association between the explanatory variables and the responses is modelled through *q* linear regressions linked by a hierarchical model. Each of the *q* regression equations is given by

$$Y_{k}-A_{k}-XB_{k}∼N(I_{n},\Sigma_{k}),$$

where the matrix of regression coefficients *B_k_* is of size p×r, whose generic element $\beta_{kj\ell }$ is the regression coefficient relating *x_j_* to *y_k_* in condition ℓ. Finally, $N(I_{n},\Sigma_{k})$ is the matrix normal distribution with independent rows, and columns correlated according to the *r* × *r* covariance matrix Σ*_k_* (Brown *et* *al.*, 1998). The intercept *A_k_* and the between-conditions covariance matrix Σ*_k_* are specific to each response *k.* To perform variable selection, we introduce a binary matrix Γ of size *q* × *p* such that $\gamma_{kj}=0$ implies $\beta_{kj\ell }=0$ for all ℓ and $\gamma_{kj}=1$ implies $\beta_{kj\ell }\neq 0$ for for all ℓ. For a given *k*, we denote by *γ_k_* the row binary vector $\left(\gamma_{k1},\ldots ,\gamma_{kp}\right)$ with the number of its non-zero entries given by $\left|\gamma_{k}\right|$. The matrix $X_{\gamma_{k}}$ of size $n\times |\gamma_{k}|$ is obtained by selecting from the matrix *X* all the columns *j* such that $\gamma_{kj}=1$. Similarly, we define $B_{\gamma_{k}}$ to be the matrix of non-zero coefficients of dimension $|\gamma_{k}|\times r$ obtained by selecting from the matrix *B_k_* all the rows *j* such that $\gamma_{kj}=1$.

Conditionally on Γ, $A_{1},\ldots ,A_{q},\;B_{1},\ldots ,B_{q}$ and $\Sigma_{1},\ldots ,\Sigma_{q}$, the *q* sparse regressions

$$Y_{k}-A_{k}-X_{\gamma_{k}}B_{\gamma_{k}}∼N(I_{n},\Sigma_{k})$$

are independent, and their joint likelihood is given by

$$\begin{array}{l} \prod_{k=1}^{q}({\left(2\pi \right)}^{r}|\Sigma_{k}|{)}^{-n/2}\exp\{-\frac{1}{2}\text{tr}[\Sigma_{k}^{-1}\times \\ {\left(Y_{k}-A_{k}-X_{\gamma_{k}}B_{\gamma_{k}}\right)}^{\text{T}}(Y_{k}-A_{k}-X_{\gamma_{k}}B_{\gamma_{k}})]\}. \end{array}$$

### 3.2 Prior settings

The prior distribution for the regression coefficients for the *k*th response is the matrix–variate normal

$$B_{\gamma_{k}}\;|\gamma_{k},g,\Sigma_{k}\;∼\;N(\;g{\left(X_{\gamma_{k}}^{T}X_{\gamma_{k}}\right)}^{-1},\Sigma_{k}\;),$$

centred in the $|\gamma_{k}|\times r$ matrix of 0 s, where the prior covariance of the regression coefficients follows a *g*-prior (Bottolo and Richardson, 2010). We choose for the constant *A_k_* the non-informative prior $p(A_{k})\propto 1$. To gain flexibility in fitting the correct amount of shrinkage, the parameter *g* is learned from the data, and its prior distribution is given by $g∼\text{InvGamma}(a_{g},b_{g})$.

For the binary matrix Γ, we introduce a *q* × *p* matrix Ω such that $\text{P}(\gamma_{kj}=1\;|\;\Omega )=\omega_{kj}$. To favour hotspot detection while maintaining a sparsity prior Γ, we decompose each cell of the matrix *ω* as $\omega_{kj}=\omega_{k}\times \rho_{j}$, where the row effect *ω_k_* accounts for the sparsity, and the column effect *ρ_j_* accounts for the propensity of predictor *j* to be a hotspot, that is to be associated to a significant proportion of the responses (see the following section for hotspots definition in practical applications). The sparsity prior is given by $\omega_{k}∼\text{Beta}(a_{\omega_{k}},b_{\omega_{k}})$ and $\rho_{j}∼\text{Gamma}(c_{\rho_{j}},d_{\rho_{j}})$, where $\omega_{1},\ldots ,\omega_{q}$ and $\rho_{1},\ldots ,\rho_{p}$ are all *a priori* independent. To enforce that *ω_kj_* is a probability, we impose the constraint $0\leq \omega_{k}\times \rho_{j}\leq 1$. The interested reader is referred to Bottolo *et* *al.* (2011) for further information about the multiplicative decomposition of *ω_kj_* and the sparsity priors assigned to its components. Finally, the prior for the cross-tissue error covariance matrix Σ*_k_* is an Inverse Wishart distribution, $\Sigma_{k}∼\text{IW}(d,h_{k}I_{r})$.

The priors we have chosen allow to integrate out the regression coefficients *B_k_*, the intercepts *A_k_* and the residual cross conditions covariances Σ*_k_.* For each response *k*, k=1,…,q, the conditional marginal likelihood is given by

$$p(Y_{k}|X,\gamma_{k},g)\propto {\left(1+g\right)}^{-r|\gamma_{k}|/2}\times {\left|h_{k}I_{n}+\frac{1}{1+g}Y_{k}^{T}Y_{k}+\frac{g}{1+g}R(\gamma_{k})\right|}^{(d+n+r-2)/2},$$

where

$$R(\gamma_{k})=Y_{k}^{\text{T}}Y_{k}-Y_{k}^{\text{T}}X_{\gamma_{k}}{\left(X_{\gamma_{k}}^{\text{T}}X_{\gamma_{k}}\right)}^{-1}X_{\gamma_{k}}^{\text{T}}Y_{k}.$$

The joint distribution of the Bayesian model factorizes as

(1) $$\left\{\prod_{k=1}^{q}p(Y_{k}\;|\;X,\gamma_{k},g)p(\gamma_{k}\;|\;\omega_{k},\rho_{1},\ldots ,\rho_{p})\;p(\omega_{k})\right\}\times p(g)p(\rho_{1},\ldots ,\rho_{p})$$

To complete the specification of the model, we need to discuss the hyperparameters setting for the priors. Regarding the shrinkage parameter *g*, a popular choice is the Zellner–Siow prior g∼InvGamma(1/2,n/2), which allows to learn the amount of shrinkage from the data. Theoretical results show good properties of this prior in model selection (Liang *et* *al.*, 2008), but little is known when the Zellner prior is used in *q* related (multiple conditions/tissues) regressions with an exchangeable shrinkage prior on *g.* In our set-up we have found empirically that when *q* is large the level of shrinkage is too low. We adapted the Zellner–Siow prior to related multiple responses by taking g∼InvGamma(q/2+q−1,nq/2). This corresponds to keep the same prior mode for *g* as in *q* = 1, and at the same time increasing the precision of the prior proportionally to the number of responses. In our experiments, this improved the performance.

We fix $c_{\rho_{j}}=1.2$ and $d_{\rho_{j}}=1.2$ for all *j*, in order to centre *ρ_j_* on average around 1. This choice of parameters for the Gamma distribution ensures a finite mode while allowing a large coefficient of variation, thus providing necessary shrinkage for most *ρ_j_*s towards 1 but also supporting some large *ρ_j_*s to enhance hot-spot detection. Alternative parameterizations cannot achieve these two competing goals simultaneously. See Supplementary Material, Section S.4, for a sensitivity analysis on a simulated and real dataset. We let the parameters $a_{\omega_{k}}$ and $b_{\omega_{k}}$ to be chosen according to the specific dataset to be analysed. These hyperparamters are determined by back calculation once, for each *k*, $\text{E}(|\gamma_{k}|$) and $\text{Var}(|\gamma_{k}|$) (respectively, the *a priori* expected number of predictors and its variance for each response *k*) are specified.

Finally, for the covariance matrices Σ*_k_*, we choose the convenient value *d* = 3, which brings $\text{E}(\Sigma_{k})=h_{k}I$. The choice for *h_k_* is more complicated, as it should be comparable in size with the likely error variance. We use the same empirical Bayes approach as in Petretto *et* *al.* (2010), that is, for a given response *k*, we run a stepwise regression for each condition ℓ=1,…,r. Then we set *h_k_* as the median of the condition-specific estimates of the error variance.

### 3.3 MCMC algorithm

The inference on this high-dimensional model is performed using a specifically designed MCMC algorithm. This algorithm has already been presented in great detail in Bottolo and Richardson (2010), Richardson *et* *al.* (2010) and Bottolo *et* *al.* (2011), so we are simply sketching its structure, and we refer to the relevant sources for all the technical details.

The variables sampled are those that appear in the posterior density (1), that is the *q* × *p* matrix $\Gamma ={\left(\gamma_{1},\ldots ,\gamma_{q}\right)}^{\text{T}}$, the vectors $\omega_{1},\ldots ,\omega_{q}$ and $\rho_{1},\ldots ,\rho_{p}$, and the parameter *g.* The challenging part is the sampling of the matrices Γ, and this problem is solved by relying on an Evolutionary Monte Carlo (EMC) structure as introduced in Liang and Wong (2000). To avoid being trapped in a local mode, EMC samples several chains, each with its own temperature, and allows them to exchange information. At each step, the chains are updated by local moves which act on a single chain (i.e. traditional MCMC moves), as well by global moves, which use a selection step inspired from genetic algorithms.

In our setting, if we run the MCMC algorithm for *T* sweeps and *C* chains, its output is given by $\Gamma^{\left(t,c\right)},\;\omega_{1}^{\left(t,c\right)},\ldots ,\omega_{q}^{\left(t,c\right)},\;\rho_{1}^{\left(t,c\right)},\ldots ,\rho_{p}^{\left(t,c\right)}$ and $g^{\left(t\right)}$ with t=1,...,T and c=1,...,C. The parameter *g* has been kept in a single chain because this setting allowed for a faster convergence of our algorithm (see Bottolo *et* *al.*, 2011). All the details regarding the local moves and the global moves, as well as the tuning of the temperature parameters, can be found in Bottolo and Richardson (2010) and Bottolo *et* *al.* (2011).

The following pseudo-algorithm shows the various moves performed at each iteration.

Algorithm 1. MCMC algorithm iteration

1.  Sample uniformly the responses to update.
2.  For each response *k* to update:
     2.1 For each chain *c*, update the *k*th row of $\Gamma^{\left(c\right)}$ by using a local move.
     2.2 Update the *k*th row of $\Gamma^{\left(1\right)},\ldots ,\Gamma^{\left(C\right)}$ by using a global move.
     2.3 Update $\omega_{k}^{\left(1\right)},\ldots ,\omega_{k}^{\left(C\right)}$.
3.  Update $\rho_{1}^{\left(1\right)},\ldots ,\rho_{p}^{\left(1\right)},\ldots ,\rho_{1}^{\left(C\right)},\ldots ,\rho_{p}^{\left(C\right)}$.
4.  Update the global shrinkage parameter *g.*

### 3.4 Post-processing

#### 3.4.1 Declaration of associations and hotspots

The posterior distribution of any function of any subset of model parameters can be calculated straightforwardly in the MCMC estimation of the model. Since the model includes explicit variable selection parameters (*γ_kj_*), we focus on functions of those parameters in order to summarize the evidence of association between predictors and responses.

Pairwise association between response *k* and predictor *j* is thus quantified by the marginal posterior probability of inclusion (MPPI)

$$\pi_{kj}=\text{P}(\gamma_{kj}=1|Y_{1},\ldots ,Y_{q},X).$$

This quantity summarizes the evidence brought by all the data regarding whether the predictor *x_j_* is associated with the n×r response *Y_k_.* It is straightforwardly estimated by the average number of times $\gamma_{kj}=1$ during the MCMC run. In order to obtain a list of associations of interest, we need a decision rule. The simplest choice is to threshold on the MPPI: declare an association if *π_kj_* is greater than some threshold value *c.* The threshold is set according to the user’s required balance between false positives and false negatives. An alternative approach is to calculate the so-called Bayesian FDR (Broët *et* *al.*, 2004; Muller *et* *al.*, 2006). For a given threshold *c*, this is defined as

$$bFDR\equiv \frac{\sum_{k,j}\left(1-\pi_{kj})I[\pi_{kj}>c\right]}{\sum_{k,j}I[\pi_{kj}>c].}$$

Users can either calculate the *bFDR* for a given threshold on the *π_kj_*, or can set a required level of *bFDR* and use that to find a threshold on the *π_kj_.* Either way, the declared pairwise associations are those with the largest values of *π_kj_.*

In the Supplementary Material, Section S.3, we give the true FDR values for our simulation datasets for the decision rule defined by setting a threshold on *bFDR.* We find that on average the method is conservative, i.e. the *bFDR* overestimates the true FDR value in line with the strong shrinkage of the *π_kj_* resulting from our sparsity assumptions. Thus, reported hotspots using *bFDR* cut-offs carry a higher level of support than their nominal level.

For the results in this article, we use the *π_kj_* to declare hotspots as well as pairwise associations. We use a target value of the *bFDR* to find a threshold on the *π_kj_* to declare pairwise associations, and subsequently count the number of associations for each predictor. Other options would be to consider directly the number of associations $\sum_{k}\gamma_{kj}$ for predictor *j*, and use the posterior mean or posterior probability of reaching a given level to declare hotspots.

Focussing on a particular response *k*, it can also be of interest to look at the whole set of predictors found to be associated with that response. For this we consider the whole vector *γ_k_.* We calculate a ‘re-normalized’ estimate of posterior model probabilities for each response, see Supplementary Material, Section S.2, for details.

#### 3.4.2. Model adequacy

In this section, we describe the Bayesian implementation of leave-one-out prediction error and model adequacy (Gelfand *et* *al.*, 1992). For ease of notation, in the following we bypass the subscript ℓ that indicates the condition/tissue under investigation. Let $\gamma_{k}^{B}=\{\gamma_{k}^{B}:B=\max_{t}p(\gamma_{k}^{\left(t\right)}|Y_{k},X)\}$ be the vector of the best model visited during the MCMC for response *k.* For each response *k*, given the best model visited $\gamma_{k}^{B}$ and after integrating out the parameter *g* numerically, we generate observations $y_{ik}^{f}$ from the predictive density $f(y_{ik}^{f}|y_{(i)k},\gamma_{k}^{B})$, where $y_{(i)k}$ denote the n−1  data vector with *y_ik_* deleted. Under model adequacy, *y_ik_* is then checked against $f(y_{ik}^{f}|y_{(i)k},\gamma_{k}^{B})$. Since, if the model holds, *Y_ik_* is a random realization of $f(Y_{ik}^{f}|Y_{(i)k},\gamma_{k}^{B})$, we use

(2) $$g(y_{ik},y_{ik}^{f})=\frac{y_{ik}-\hat{\text{E}}(Y_{ik}^{f}|y_{(i)k},\gamma_{k}^{B})}{\sqrt{\hat{\text{Var}}(Y_{ik}^{f}|y_{(i)k},\gamma_{k}^{B})}}$$

as checking function where $\hat{\text{E}}(Y_{ik}^{f}|y_{(i)k},\gamma_{k}^{B})$ and $\hat{\text{Var}}(Y_{ik}^{f}|y_{(i)k},\gamma_{k}^{B})$ are the empirical mean and variance calculated from the MCMC iterations. For each response, squared prediction error is summed over the sample and the quantity $\sum_{i}{\left[g(y_{ik},y_{ik}^{f})\right]}^{2}$ is then used as a measure of model adequacy with large values indicating that the model performs poorly. This quantity resembles the widely adopted mean-square error with the notably modification that now the differences between the predicted and observed values are standardized.

### 3.5 Simulation study

In order to investigate the performance of our multi-tissue method, we construct an artificial dataset with three tissues and 150 responses. As in Bottolo *et* *al.* (2011), we use the whole genome data of rat Recombinant Inbred (RI) strains derived from a cross between the Spontaneously Hypertensive Rat (SHR) and the Brown Norway (BN) strains, which we have used previously to identify eQTLs across multiple tissues (Heinig *et al.*, 2010; Hubner *et* *al.*, 2005; Petretto *et* *al.*, 2006). This choice allows us to test the performance of our algorithm and compare it with alternatives methods when complex patterns of correlation between markers depends on genetic forces that shape the structure of LD. After removing redundant variables, the genome-wide number of SNPs is reduced to 1304, giving a 29×1304 *X* matrix.

Conditionally on the SNP matrix *X*, we generate responses for each tissue from a linear model. For each tissue ℓ, 1≤ℓ≤3, the *n* × *q* matrix $Y_{\ell }$ gathering the observations of the 150 responses is given by the regression

$$Y_{\ell }=XB_{\ell }+E_{\ell }+E^{\text{shared}},$$

where $B_{\ell }$ is the *p* × *q* matrix of regression coefficients, $E_{\ell }$ is the *n* × *q* matrix of residuals for tissue ℓ and $E^{\text{shared}}$ is a *n* × *q* matrix of residuals shared by all tissues. The residuals are given by $\epsilon_{ik\ell }∼N(0,\sigma_{\ell }^{2})$ and $\epsilon_{ik}^{\text{shared}}∼N(0,\sigma_{\text{shared}}^{2})$.

**Fig. 1.**
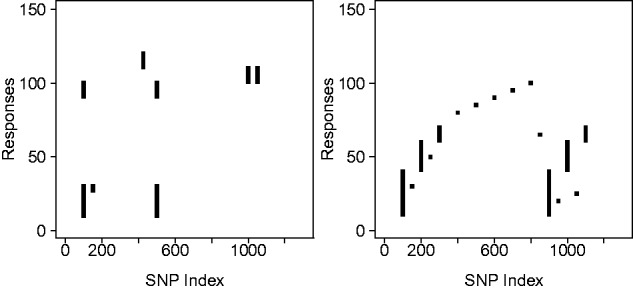
Matrix Γ for the pattern of associations common to the three tissues. The entry (*k*, *j*) of the matrix Γ indicates the presence of an association between SNP *j* and response *k* in all tissues. (**a**) Main simulation study. (**b**) Study for distinguishing *cis*- and *trans*-associations

The matrix $B_{\ell }$ controls the pattern and strength of signal between each responses and predictors in tissue ℓ. For the simulations we keep $B_{\ell }$ constant across tissues ($\beta_{kj\ell }=\lambda_{kj}\times \gamma_{kj}$) and control the signal-to-noise patterns through the noise variance parameters. The pattern of non-zero associations between responses and predictors is encoded by the matrix Γ of 0 s and 1 s. Because, our method is aimed at detecting a signal that is present in different conditions, we use the same association pattern Γ in all conditions. This configuration can be seen in Figure 1 where six hotspots (vertical bars) with a varying number of associations per hotspot are simulated. The exact specification is given in the Supplementary Material, Section S.1.1. The signal strength is parameterized by *μ*, with $\lambda_{kj}∼N(\mu ,0.001^{2})$.

The signal-to-noise ratio in each tissue is tuned thorough the tissue-specific noise standard deviation $\sigma_{\ell }$ and the ‘shared’ noise standard deviation $\sigma_{\text{shared}}$. Assuming without loss of generality independence of the noise variances, the total noise standard deviation for tissue ℓ is given by $\sigma_{\ell }^{\text{total}}=\sqrt{\sigma_{\ell }^{2}+\sigma_{\text{shared}}^{2}}$. The correlation between tissues is proportional to the ‘shared’ noise variance and also depends on the noise variances of the two tissues considered (see Supplementary Material, Section S.1.2).

We have conducted an extensive investigation into the effect of the signal-to-noise ratio imbalance across tissues and here we show the output of the simulations for independent residuals ($\sigma_{\text{shared}}=0$) and correlated residuals ($\sigma_{\text{shared}}=0.04$). In each case, we consider a balanced case with $\sigma_{\ell }^{\text{total}}=0.1,\;1\leq \ell \leq 3$, and an unbalanced case with $\left\{\sigma_{1}^{\text{total}}=0.1,\sigma_{2}^{\text{total}}=0.2,\sigma_{3}^{\text{total}}=0.4\right\}$. We also ran the simulations with several levels of the signal strength *μ.* For the plots presented here we chose μ=0.15.

For each simulation set-up we generated nine replicates, and for each of the datasets simulated, we ran MT-HESS analysing the three tissues jointly and a MANOVA test (Nath and Pavur, 1985) for each multiple tissue response–SNP pair separately. We also include the receiver operating characteristic (ROC) curves intersecting the results from the single-tissue version of HESS run on each separate tissue (following Fairfax *et* *al.*, 2012). We call these analyses iST-HESS, intersection of single tissue HESS, to distinguish it from MT-HESS, multi-tissue HESS. Associations were declared by thresholding MPPI values for MT-HESS and ST-HESS and *P*-values for MANOVA. For the intersection iST-HESS, associations are declared positive for a given threshold if the single-tissue MPPIs exceed the threshold in all three tissues.

**Fig. 2.**
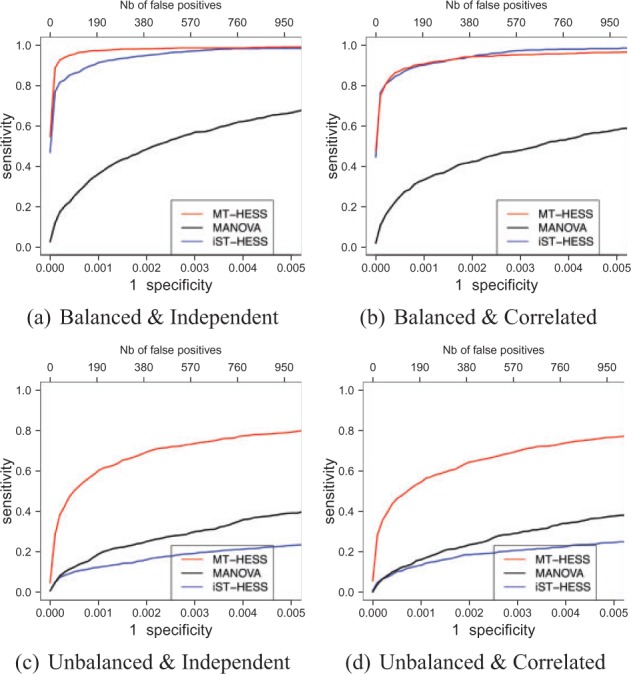
ROC curves comparing results of MT-HESS, MANOVA and iST-HESS. Top row plots have balanced across tissues total noise standard deviation $\sigma_{\ell }^{\text{total}}=0.1,\;1\leq \ell \leq 3$, bottom row are unbalanced with total noise standard deviation $\left\{\sigma_{1}^{\text{total}}=0.1,\sigma_{2}^{\text{total}}=0.2,\sigma_{3}^{\text{total}}=0.4\right\}$. Left hand column plots have uncorrelated residuals between tissues with $\sigma_{\text{shared}}=0$, right hand column have $\sigma_{\text{shared}}=0.04$

Figure 2 shows ROC curves for each of the four combination of balanced/unbalanced noise across tissues and independent/correlated residuals between tissues. We see that MT-HESS clearly outperforms MANOVA in all scenarios, which confirms what has already been shown in the literature, that is penalization methods outperform ‘one-at-a-time’ methods (Bottolo *et* *al.*, 2013), and that borrowing information across responses improves even further the performance (Richardson *et* *al.*, 2010; Scott-Boyer *et* *al.*, 2012).

Comparison between MT-HESS versus iST-HESS also confirms previous results (Petretto *et* *al.*, 2006, 2010) that the intersection of tissue-specific findings is likely to be a conservative approach and is potentially affected by variability between tissues. Specifically in the unbalanced case, MT-HESS strongly outperforms the intersection of separate single-tissue results. This is still evident, although less pronounced, in the balanced case with independent noise. As expected, correlated noise lower slightly the ROC curves for the multi-tissue methods (MT-HESS and MANOVA) since it encourages propagation of false positives across all the tissues. Obviously, this does not affect iST-HESS. Note that the balanced cases have higher signal-to-noise ratio than the unbalanced ones, and so ROC curves for all methods are higher.

Supplementary Material, Section S.1, shows a greater range of simulation set-ups and detailed findings for all the methods compared here, i.e. MT-HESS, MANOVA and iST-HESS. In particular, we provide further results obtained by specifying different levels of the signal strength *μ.* We illustrate how MT-HESS achieves a better separation between noise and signal and how it allows to identify more hotspots than traditional methods.

We now investigate the ability of the model to detect different types of association patterns. Since the model is designed to borrow information across responses, it is of interest to see whether the model can also detect predictors associated with a single response, for instance, if we can still detect *cis*-eQTLs. To look at this, we use a different pattern of associations, with six markers designated as hotspots (two each with 10, 20 and 30 responses associated) and 10 *cis* markers. Half the *cis*-associations are completely isolated, for the other half the response is also associated with other (*trans*) markers. The pattern is shown in Figure 1b. We allow the *cis*-associations to be stronger than those in the hotspots, as *trans*-associations are expected to be weaker, thus we now have two parameters for signal strength $\mu_{\text{cis}}=0.6$ and $\mu_{\text{trans}}=0.15$ with independent residuals ($\sigma_{\text{shared}}=0$) and balanced total noise standard deviation $\sigma_{\ell }^{\text{total}}=0.1,\;1\leq \ell \leq 3$. ROC curves for these associations are very similar to the case shown in Figure 2a (data not shown). The overall performance for all methods considered here is slightly lower for the new *cis*- and *trans*-association pattern, due to the lower proportion of associations contained in hotspots.

To compare in detail *cis*- and *trans*-results, we classify the response–predictor pairs as ‘true negative’, ‘true *cis* isolated’, ‘true *cis* other’ and ‘true *trans*’ (part of hotspot). Table 1 shows the cross-classification of ‘true’ and ‘called’ status for the three approaches considered here. Thresholds for calling pairwise associations were determined by fixing the true FDR level at 10%. We see that while both MT-HESS and iST-HESS are able to detect the *cis*-associations very well, the multiple tissue model is able to pick up more of the associations that are contained in hotspots, thus showing the benefit of combining the tissues in a fully Bayesian way. The MANOVA misses all hotspot associations in this example. Looking directly at the hotspots, Table 2 shows the average estimated size of each true hotspot (defined as number of responses called marginally): it is clear that MT-HESS has more power to detect hotspots than iST-HESS.

**Table 1.** Classification table for marginal pairwise associations at a true FDR level of 10%

	True negative	True *cis* isolated	True *cis* other	True *trans*
MT-HESS				
Negative call	195 458.2	0.0	0.0	28.2
Positive call	11.8	5.0	5.0	91.8
MANOVA				
Negative call	195 469.0	0.6	4.4	120.0
Positive call	1.0	4.4	0.6	0.0
iST-HESS				
Negative call	195 462.3	0.0	0.0	63.2
Positive call	7.7	5.0	5.0	56.8

True positives are split into ‘true *cis* isolated’, ‘true *cis* other’ and ‘true *trans*’ associations. Numbers are averages over nine replications

**Table 2.** Average size of hotspots declared at a true FDR level of 10% on pairwise associations of 10%

True size	10	20	30
MT-HESS	5.8	17.3	24.3
MANOVA	0.0	0.0	0.0
iST-HESS	1.4	10.7	16.3

Numbers are averages over nine replications

## 4 Results

### 4.1 Rat data

The rat dataset consists of multiple tissue expression and genome-wide genotype data (∼1400 SNPs) in a panel of 29 RI rat strains (Petretto *et* *al.*, 2006). Here, we focus on the multi-tissue eQTL mapping of a previously reported Interferon regulatory factor 7 *IRF7*-driven inflammatory network (IDIN) found to be associated with immune response and increased risk of type 1 diabetes in humans (Heinig *et* *al.*, 2010).

We used HESS to re-analyse a sub-set of the IDIN network in three rat tissues (left ventricle, aorta and liver) focusing on 146 probe sets (corresponding to 143 protein coding genes), which are both robustly expressed and varying in expression across the three tissues. See Supplementary Material, Section S.5, for details of how these genes were chosen. We ran ST-HESS separately in each tissue and MT-HESS simultaneously across the three tissues, as well as a MANOVA (Nath and Pavur, 1985) analysis.

**Fig. 3.**
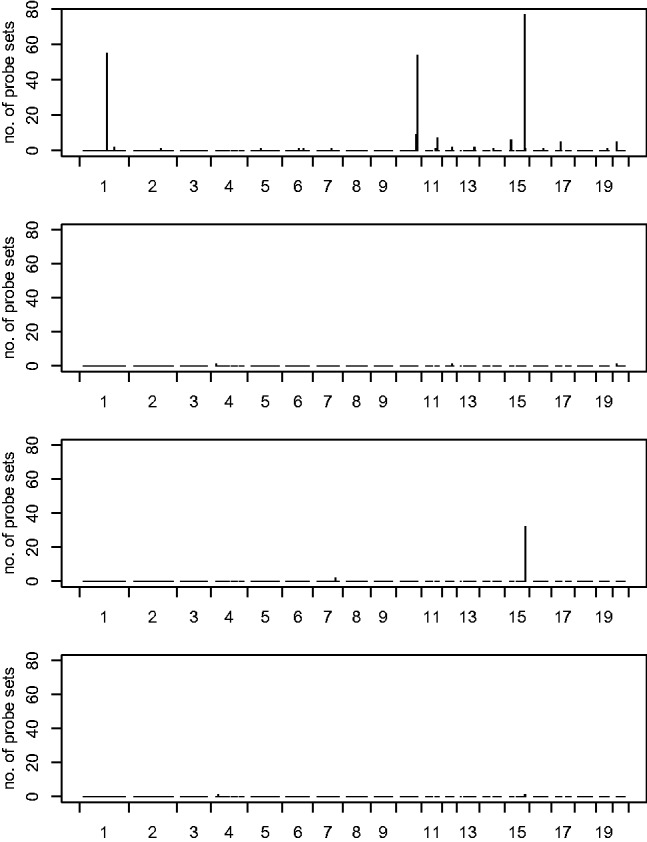
Genome-wide Manhattan-like plots for the rat analysis showing the number of probe sets in the IDIN network associated with each SNP according to genomic location. The *x*-axis depicts chromosome number and position. The *y*-axis shows the number of probe sets significantly associated with each SNP (bFDR ≤0.05). From top to bottom: results for MT-HESS, and for ST-HESS in aorta, left ventricle and liver

We ran HESS on a 2.90-GHz Intel(R) Xeon(R) CPU computer with 130 GB of memory for 15 000 sweeps of which 5000 as burn-in and the same hyper-parameters as in the simulation studies. Computational time was 70 and 114 min for ST-HESS (average across tissues) and MT-HESS, respectively. Visual inspection of the trace of the parameters *w_k_* and *ρ_j_* and *g* show no erratic behaviour. Convergence is reached after few iterations due to the combined effect of the automatic tuning of the proposal density in the Metropolis–Hastings algorithms and the parallel chains implementation which allow good mixing. Focusing on probe set-marker associations significant when using a Bayes FDR (bFDR) of 5% (which corresponds to a MPPI > 0.8), MT-HSSS identified three loci controlling more than 30% of the probe sets under analysis (Fig. 3). In addition to the regulatory hotspot in rat chromosome 15q25, which was previously identified and experimentally validated (Heinig *et* *al.*, 2010), joint analysis of the three tissues revealed two new regulatory hotspots for the IDIN, located at marker J343641 on rat chromosome 10, and at marker SHRSPc66a05_r1_451 on rat chromosome 1, respectively. We also investigated the associations of IDIN network in each single tissue. ST-HESS analysis uncovered a signal for the rat chromosome 15q25 locus in left ventricle tissue, as previously shown (Heinig *et* *al.*, 2010), but failed to identify the new regulatory loci in chromosome 10 and 1, see Figure 3. In view of the variability of ST-HESS between the three tissues shown in Figure 3, we did not consider iST-HESS further as it lacks power in unbalanced cases.

Note that with the same 5% FDR threshold on *P*-values for declaring associations, the MANOVA analysis does not return any SNPs associated with five or more genes. In order to see if a MANOVA analysis shows some of the evidence for the hotspots discovered by MT-HESS, we also computed for each locus the sum over all transcripts of the negative log *P*-values for the MANOVA test (Supplementary Figure S16). This plot is very noisy, but it is clear that the *P*-values contain evidence for the hotspots on chromosomes 10 and 15.

Given the different output produced by MT-HESS and ST-HESS, we have also investigated their model adequacy (Gelfand *et* *al.*, 1992). We compared the two models on the basis of their leave-one-out prediction accuracy by removing one observation at a time and using the remaining observation for calculating the prediction error. Following Gelfand *et* *al.* (1992) we took a fully Bayesian perspective employing predictive distributions to evaluate model performance by comparing model prediction with what has been observed. To be precise, we use standardized prediction error as checking function, as shown in Equation (2), with squared prediction error summed over the sample as model adequacy.

Table 3 shows the value of model adequacy averaged across probe sets. The best (shared across tissues) models $\left\{\gamma_{1}^{B},\ldots ,\gamma_{q}^{B}\right\}$ obtained by MT-HESS have on overage smaller prediction error than the best models identified by ST-HESS in each tissue separately. In particular MT-HESS is able to predict better than ST-HESS in aorta and liver tissues where ST-HESS was unable to identify any hostpost. In the left ventricle tissue, the additional two hotspots detected by MT-HESS permit a more accurate prediction than the single ST-HESS hotspot. Altogether, the vast majority of probe sets are better predicted by MT-HESS: among the 32 (22%) probe sets better predicted by ST-HESS only two were declared associated at 5% bFDR with the hotspots found by MT-HESS, showing that almost all probe sets (51/53) controlled by MT-HESS multiple hotspots are better predicted than by single-tissue analysis. Overall, we conclude that the model that comprises the experimentally validated and previously undiscovered putative hotspots is a more adequate model to explain the joint variation of the transcripts across tissues.

**Table 3.** Leave-one-out prediction error of MT-HESS and ST-HESS conditionally on their best models visited

	MT-HESS	ST-HESS	MT-HESS < ST-HESS
Aorta	22.85 (3.18)	33.06 (4.49)	143 (98%)
Left ventricle	35.22 (20.31)	40.86 (15.80)	114 (78%)
Liver	23.97 (4.34)	36.88 (5.67)	143 (98%)

For each tissue, average model adequacy measure across probe sets is reported with standard deviation in brackets. Out of 146 transcripts analysed in the IDIN network, the number of times ST-HESS model adequacy measure is greater than the MT-HESS one is reported in the last column with % in brackets

We comment in more details on the regulatory hotspot located in rat chromosome 10 (rat marker J343641), which showed association with 53 probe sets (MPPI > 0.8), representing 53 protein coding distinct genes. Enrichment analysis of transcription factor binding sites (TFBSs) in the putative promoter of the corresponding human orthologous genes using PASTAA (Roider *et* *al.*, 2009) revealed over-representation of TFBS motifs for *IRF7* (adjusted *P* $=3.75\times 10^{-4}$), *IRF2* (adjusted *P* $=2.60\times 10^{-2}$) and *NFKB* (adjusted *P* $=3.19\times 10^{-2}$) and *IRF1* or *IRF10* (adjusted *P* $=3.20\times 10^{-2}$), in keeping with the known IRF-driven regulation of IDIN network genes (Heinig *et* *al.*, 2010).

Examination of the genes in the LD block around the regulatory locus on rat chromosome 10 ($r^{2}>0.8$), revealed only one annotated protein coding gene, *Foxk2*, with deletion of an exon and an intron in the Spontaneous Hypertensive Rat compared with the BN rat (Atanur *et* *al.*, 2010). *Foxk2* encodes the transcription factor forkhead box protein K2. The human orthologue *FOXK2* gene is an inhibitor of the Sendai virus-induced IFN-*β* production and, at the protein level, it forms complexes with two known interferon transcriptional repressors, *IRF2* (one of the transcription factors whose binding motifs were enriched as described above) and *IRF4* (Li *et* *al.*, 2011a). In view of this, we propose *Foxk2* as a candidate master regulator of the subset of the IDIN network genes whose expression is associated with the regulatory loci located in rat chromosome 10.

### 4.2 Human data

The human dataset comprised genotype and corresponding expression data from three purified leukocyte subsets (monocytes, and CD4 and CD8 T cells) isolated from the peripheral blood of 59 patients with inflammatory bowel disease (see Supplementary Methods for full details of the cohort). Note that CD4 and CD8 T cells are both T lymphocyte subsets, whereas monocytes are from the myeloid lineage. We ran HESS separately on each cell type, and simultaneously on the joint dataset. The analysis was restricted to 21 788 SNPs on chromosome 5 and 3248 probesets selected by highest variance (see Supplementary Material, Section S.6.1) for computational feasibility. HESS was run using a burn-in of 4000 sweeps and values recorded for 8000 sweeps. MT-HESS took 110 h on a 2.80-GHz Intel(R) Xeon(R) computer.

**Fig. 4.**
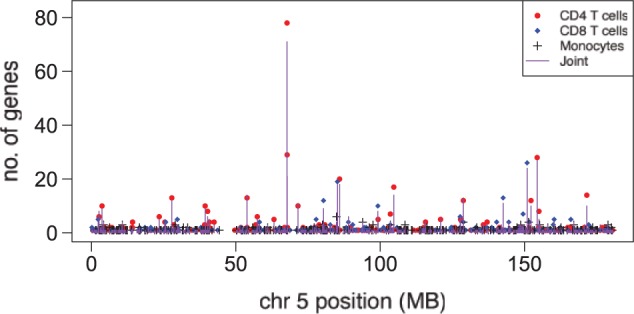
Manhattan plot for hotspot SNPs. The plot shows the number of genes significantly associated with each SNP according to position on chromosome 5 using an MPPI threshold corresponding to a 5% Bayes FDR. Vertical purple lines indicates results from the joint analysis. Red circles, blue diamonds and black crosses indicate single tissue analysis in CD4 T cells, CD8 T cells and monocytes, respectively

In the single cell type analysis, hotspots were most frequently detected in CD4 T cells, followed by CD8 T cells, whereas in monocytes we found a paucity of hotspot SNPs (Fig. 4). Using a 5% bFDR, the maximum number of genes associated with a SNP in monocytes was 6, versus 78 in CD4 T cells. There was less gene–gene correlation in the monocyte expression data (Supplementary Fig. S.17a–c), and this may account for the lack of hotspot detection in this cell type.

In the joint MT-HESS analysis 28 SNPs were associated with expression of five or more genes at a 5% bFDR significance threshold, with four of these SNPs associated with 20 or more genes (Supplementary Fig. S.18). The signal in the joint analysis appears to be driven predominantly by signal in either the CD4 T cells or the CD8 T cells (Fig. 4). Unlike in the analysis of the rat data, we did not observe any hotspots that were not detected in the single tissue analysis. However, the number of genes associated with hotspots in the MT-HESS analysis was not substantially diminished despite the hotspots being highlighted mostly in one of the three cell-types (Supplementary Fig. S.18).

We identified a putative *trans* master regulator SNP, rs11745891, associated with the highest number of genes in both the CD4 T cell and the joint analysis (78 and 71 genes, respectively). The SNP lies in an intergenic region, ∼143 kB downstream from the end of the nearest gene, *PIK3R1* (phosphoinositide-3-kinase, regulatory subunit 1 (alpha)). Histone marks from ENCODE (Dunham *et* *al.*, 2012) suggest the SNP lies near a regulatory locus (Supplementary Fig. S.19). Interestingly, this hotspot SNP lies 10.7 kb from the start site of a long intergenic non-coding (linc) RNA (Ensembl transcript *ENST00000507733*). Emerging evidence suggests that long non-coding RNAs play an important role in orchestrating transcriptional programmes through a variety of mechanisms, commonly involving ribonucleic acid–protein interactions (Rinn and Chang, 2012). We hypothesize that our SNP acts in *cis* on the lincRNA, and thus exerts its *trans* effects through differential expression of the lincRNA according to genotype.

The 78 genes whose expression in CD4 T cells was associated with rs11745891 genotype are located on 21 chromosomes, most frequently chromosome 1 (nine genes) (Supplementary Fig. S.20). *P*-values from simple univariate regression of the expression of these genes on rs11745891 were skewed towards zero (range from $1.23\times 10^{-3}$ to $1.57\times 10^{-8}$, see Supplementary Fig. S.21). However, after multiple-testing of 3248×217 888=70 767 424 SNPgene pairs, using a 5% FDR threshold (Benjamini–Hochberg procedure), none remained statistically significant, demonstrating the strength of HESS in high-dimensional omics analysis.

**Fig. 5.**
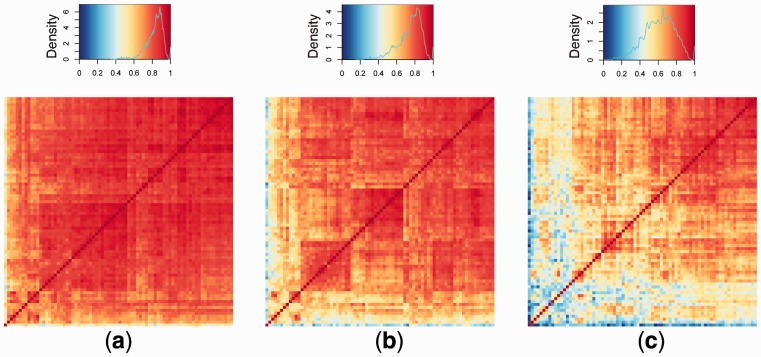
(**a**) Correlation matrix of expression of the 78 genes associated with hotspot SNP rs11745891 in CD4 T cells. (**b**) Correlation matrix of the residuals after simple regression of expression of each gene on rs11745891 genotype. (**c**) Correlation matrix of the residuals after regression of each gene on the SNPs in its best model. All three correlation matrices have been hierarchically clustered. Colour key and distribution of Pearson correlation coefficients are shown in the upper panels

The expression levels of these 78 genes were very highly correlated (Fig. 5a). Strong correlation persisted in the residuals from the simple regression of expression of these genes on rs11745891 genotype (Fig. 5b). A strength of HESS over traditional one SNP at-a-time methods is its multivariate approach combined with variable selection, allowing us to ask what *combination of SNPs* best explain gene expression. Using this information, we next performed regression of expression of each gene on its best combination of SNP(s). The resulting residuals were substantially less correlated than the residuals from regression on just the hotspot SNP alone (Fig. 5c). Thus, we see that including multiple SNPs in the model accounts for a substantial fraction of the correlation in gene expression. This highlights a major advantage of our approach.

Additional discussion of the human results is given in the Supplementary Material.

## 5 Discussion

The availability of data collected over multiple tissues or conditions is becoming increasingly common given the decreasing cost of generating transcriptomic, metabolic and epigenetic phenotypes over large cohorts of individuals (Li *et* *al.*, 2011b).

In this article we present a new model that exploits the multidimensional nature of these data and extends ST-HESS presented in Bottolo *et* *al.* (2011): our new hierarchical model and C++ algorithm, MT-HESS is able to analyse a large number of responses collected over dependent multiple tissues or conditions and regress them on a large set of correlated predictors. Similar to its parent version, MT-HESS retains the ability to perform hotspot discovery in -omics experiments. To the best of our knowledge, MT-HESS is the first tool able to unravel whether a genetic marker has a systemic role and influences several OMICS traits in multiple related conditions at the same time.

This is demonstrated in the complex multiple tissue scenario of our real rat data analysis. The increased power of MT-HESS allowed the detection of both experimentally validated and previously undiscovered putative hotspots for the IDIN network across left ventricle, aorta and liver tissues. Prediction error analysis confirms the importance of the new detected hotspots. Model adequacy measure shows the superiority of MT-HESS model over ST-HESS and the benefit of the integrated analysis. While in the rats dataset it is possible to perform a leave-one-out cross-validation approach, in larger datasets out-of-sample prediction error can be performed by taking a random split of the data, for instance 50/50, and reserving the remaining data for calculating the average prediction error.

Due to a lack of competing algorithms that can analyse multiple layers of correlated responses in a fully multivariate fashion, we have compared MT-HESS with a MANOVA test, which is extensively used when multiple outcomes are present. Recall that such MANOVA test is performed for each pair of (SNP × response vector) at a time, where the vector of responses correspond to the expression in multiple tissues or conditions. We see that MT-HESS outperforms MANOVA, demonstrating that our hierarchical modelling approach is more powerful than pairwise methods.

When faced with an eQTL analysis task which involves multiple tissues, a natural alternative to MANOVA test is to compare results from the intersection of single-tissue model (iST-HESS) with those from the multiple-tissue one (MT-HSSS). Our extensive simulation study show that when there is a similar pattern of association but varying levels of signal-to-noise ratio across tissues, MT-HESS clearly outperforms iST-HESS. This is well in keeping with the construction of the model underlying MT-HESS, which performs a statistically sound evidence synthesis for detecting hotspots between the set of conditions or tissues. When signal-to-noise ratio is constant across conditions/tissues MT-HESS results become closer to those of iST-HESS pointing to the benefit of adjusting for external sources of correlated noise prior to MT-HESS analysis, for example by using a latent factor approach as in Stegle *et* *al.* (2012).

In our simulation study we show a substantial gain of power of MT-HESS when the association in each tissue were too weak to be detected by ST-HESS, a situation also illustrated in our first case study where two new hotspots were highlighted by MT-HESS, whereas they were not detected in the single tissue analyses. Here the ST-HESS signal is relatively noisy and the ability of MT-HESS to borrow and synthesize information between different layers of the responses results in higher power for MT-HESS.

Our new C++ implementation of the complex hierarchical structure in MT-HESS (comprising ST-HESS as a special case) permits efficient analysis of very large datasets. It made feasible an MT-HESS analysis of our human dataset, where a multivariate analysis and variable selection between 3000 responses in three cell types and nearly 22 000 SNPs was carried out. The putative novel *trans* master regulator situated near a long intergenic non-coding (linc) RNA (Ensembl transcript *ENST00000507733*) (see Section 4.2) is a good example of the power of our approach for generating hypotheses which can be validated by follow-up experiments. The clear separation between noise and signal offered by our sparse regression approach is paramount for successfully prioritizing such further validation.

In conclusion, our new algorithm MT-HESS with its efficient C++ architecture is tailored to jointly analyse realistic case studies that comprise a large number of responses collected over dependent multiple tissues or conditions and a large set of potentially collinear predictors.

## Supplementary Material

Supplementary Data
