# Supplementary material for: MT-HESS: an efficient Bayesian approach for simultaneous association detection in OMICS datasets, with application to eQTL mapping in multiple tissues
Source: Bioinformatics. 2015 Oct 26;32(4):523–32. doi: 10.1093/bioinformatics/btv568 (PMC4743623; doi:10.1093/bioinformatics/btv568)
Supplement: Supplementary Data [file supp_32_4_523__index.html]

MT-HESS: an efficient Bayesian approach for simultaneous association detection in OMICS datasets, with application to eQTL mapping in multiple tissues — MT-HESS: an efficient Bayesian approach for simultaneous association detection in OMICS datasets, with application to eQTL mapping in multiple tissues — Supplementary Data 

# MT-HESS: an efficient Bayesian approach for simultaneous association detection in OMICS datasets, with application to eQTL mapping in multiple tissues

## Supplementary Data

files

- Supplementary Data - pdf file
