# Supplementary material for: MT-HESS: an efficient Bayesian approach for simultaneous association detection in OMICS datasets, with application to eQTL mapping in multiple tissues
Source: Bioinformatics. 2015 Oct 26;32(4):523–32. doi: 10.1093/bioinformatics/btv568 (PMC4743623; doi:10.1093/bioinformatics/btv568)
Supplement: Supplementary Data [file supp_btv568_HESS_SUPPL_RESUBMIT.pdf]

MT-HESS: an efficient Bayesian approach for simultaneous association detection in OMICS datasets, with application to eQTL mapping in multiple tissues

Alex Lewin, Habib Saadi,
James Peters, Aida Moreno-Moral,
James Lee, Kenneth Smith, Enrico Petretto,
Leonardo Bottolo and Sylvia Richardson

Contents

S.1 Simulation study **2**
- S.1.1 Simulation set-up . . . 2
- S.1.2 Simulation study results: residual correlations between tissues . . . 3
- S.1.3 Simulation study results: unbalanced noise across tissues . . . 4
- S.1.4 Simulation study results: unbalanced noise across tissues and residual correlation between tissues . . . 5
- S.1.5 Simulation study results: strength of association signal . . . 10

S.2 Post-processing for model probabilities **16**

S.3 Calibration of FDR estimate **17**

S.4 Sensitivity analysis **18**

S.5 Rat data results **21**

S.6 Human data results **22**
- S.6.1 Human data methods . . . 22
- S.6.2 Additional discussion . . . 23
- S.6.3 Additional figures . . . 24

S.1 Simulation study

S.1.1 Simulation set-up

The matrix Γ of 0's and 1's encodes the pattern of non-zero associations between responses and predictors. Because our method is aimed at detecting a signal that is present in different tissues, we use the same Γ in all tissues.

The association pattern used in the main simulation study can be visualized in Figure 1 (a) in the main text. It contains 6 hotspots, with a varying number of associations per hotspot. The 6 hotspots are placed at the SNP position $\{100, 150, 425, 500, 1000, 1050\}$. The responses associated to each hotspot are as follows:

- **Hotspot 1**: Responses 10 to 30, and 91 to 100;
- **Hotspot 2**: Responses 27 to 30;
- **Hotspot 3**: Responses 111 to 120;
- **Hotspot 4**: Responses 10 to 30, and 91 to 100;
- **Hotspot 5**: Responses 101 to 110;
- **Hotspot 6**: Responses 101 to 110.

The pattern used to distinguish between *cis*- and *trans*-associations contains 6 hotspots, at SNP positions $\{100, 200, 300, 900, 1000, 1100\}$, 5 isolated *cis* SNPs at positions $\{400, 500, 600, 700, 800\}$ and 5 *cis*-associations which predict responses also predicted by other SNPs at positions $\{150, 250, 850, 950, 1050\}$. It can be visualized in Figure 1 (b). The responses associated are as follows:

- **Hotspot 1**: Responses 11 to 40;
- **Hotspot 2**: Responses 41 to 60;
- **Hotspot 3**: Responses 61 to 70;
- **Hotspot 4**: Responses 11 to 40;
- **Hotspot 5**: Responses 41 to 60;
- **Hotspot 6**: Responses 61 to 70;
- ***Cis* isolated**: Responses {80, 85, 90, 95, 100};
- ***Cis* other**: Responses {30, 50, 65, 20, 25}.

The most general pattern of noise across the tissues in our simulation study is as follows:

$$\epsilon_{ik1} \sim \mathrm{N}(0, \sigma_1^2)$$
$$\epsilon_{ik2} \sim \begin{cases} \mathrm{N}(0, \sigma_{21}^2) & k \notin [91:120] \\ \mathrm{N}(0, \sigma_{22}^2) & k \in [91:120] \end{cases}$$
$$\epsilon_{ik3} \sim \begin{cases} \mathrm{N}(0, \sigma_{31}^2) & k \notin [100:120] \\ \mathrm{N}(0, \sigma_{32}^2) & k \in [100:120] \end{cases}$$
$$\epsilon_{ik}^{\text{shared}} \sim \mathrm{N}(0, \sigma_{\text{shared}}^2)$$

This is visualised in Figure S.4, along with the coefficient of determination R^2 plot for a case with unbalanced total noise standard deviation across tissues.

S.1.2 Simulation study results: residual correlations between tissues

In Figure S.1 we show Receiver Operating Characteristic (ROC) curves of MT-HESS and MANOVA for three simulation set-ups with equal total noise standard deviation $\sigma_\ell^{\text{total}} = 0.1$, $1 \leq \ell \leq 3$, but with increasing correlation between tissues (0, 0.16 and 0.5). The correlation between tissues is proportional to the "shared" noise variance and also depends on the noise variances of the two tissues considered. Given two tissues ℓ and ℓ', the correlation between them is given by

$$\rho_{\ell\ell'} = \frac{\sigma^2_{\text{shared}}}{\sqrt{\sigma^2_\ell + \sigma^2_{\text{shared}}}\sqrt{\sigma^2_{\ell'} + \sigma^2_{\text{shared}}}}.$$

In the balanced case, when the tissue-specific noise standard deviation is constant across tissues, the equation above simplifies with constant level of correlation between tissues. In this case, without loss of generality, we drop the subscripts ℓ, ℓ'. We also report ROC curves for individual tissues. We call these analyses ST-HESS, single tissue HESS, to distinguish it from MT-HESS, multi-tissue HESS. Although ST-HESS ROC curves are not properly comparable to those from MT-HESS ones, as they result from different subsets of the data, they are included to give some insight into the way in which the multiple tissues methods combine information across the tissues. Since we keep the total noise standard deviation constant in all three cases, each single-tissue ROC curves remain the same as they are not influenced by the level of correlation. Finally we have also added the ROC curves intersecting the results from the single-tissue version of HESS run on each separate tissue. We call these analyses iST-HESS, intersection of single tissue HESS, to distinguish it from single and multiple tissues analysis above. iST-HESS ROC curves do not depend on the level of correlation between tissues and therefore remain the same for all the cases simulated. As expected iST-HESS analysis is more powerful at detecting associations than any single-tissue analysis. In contrast we see that MT-HESS gains less power over the single tissue analysis as the correlation between tissues increases. MANOVA, which also uses an estimate of the covariance across tissues is also affected by the correlation level.

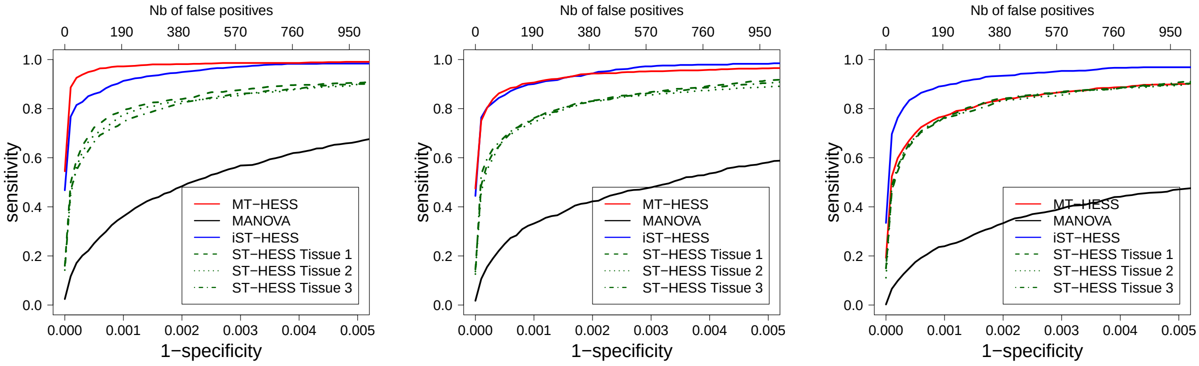

(a) $\rho = 0$ (b) $\rho = 0.16$ (c) $\rho = 0.50$

Figure S.1: ROC curves of MT-HESS, MANOVA, iST-HESS and tissue specific ST-HESS for simulations with balanced total noise standard deviation across tissues, $\sigma_\ell^{\text{total}} = 0.1$, $1 \leq \ell \leq 3$ and level of the signal strength $\mu = 0.15$. Plots have different levels of correlation between tissues due to different levels of tissue-specific noise standard deviation and "shared" noise standard deviation: (a) $\sigma_\ell = 0.10$ & $\sigma_{\text{shared}} = 0$, (b) $\sigma_\ell = 0.092$ & $\sigma_{\text{shared}} = 0.040$, (c) $\sigma_\ell = 0.071$ & $\sigma_{\text{shared}} = 0.071$.

S.1.3 Simulation study results: unbalanced noise across tissues

We have also investigated a range of simulation cases with different signal-to-noise ratios across tissues with the same level of the signal strength $\mu = 0.15$ as before. Here we show cases with independent residuals between tissues, in order to separate out the effects of imbalanced noise standard deviation in the three tissues and correlation between tissues. Figure S.2 shows the ROC curves for four simulation cases, with varying values of signal-to-noise ratio across the three tissues. The individual ST-HESS curves for the different tissues give an indication of the amount of imbalance of signal-to-noise ratio across the three tissues. The three methods which combine results across tissues (MT-HESS, MANOVA and iST-HESS) in general lie in between the separate tissue curves, as they all perform an averaging across tissues. We see clearly the advantage of MT-HESS over both MANOVA and the intersection of single-tissue results.

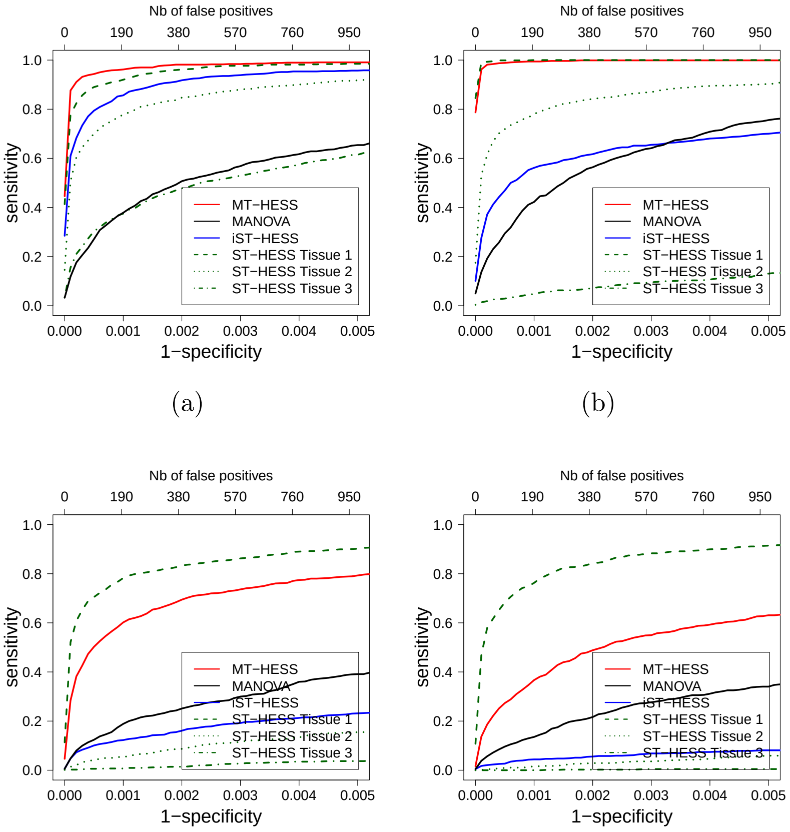

(a) (b) (c) (d)

Figure S.2: ROC curves of MT-HESS, MANOVA, iST-HESS and tissue-specific ST-HESS with independent residuals between tissues and unbalanced tissue-specific noise standard deviations. Level of the signal strength $\mu = 0.15$. Tissue-specific noise standard deviation: (a) $\sigma_1 = 0.8, \sigma_2 = 0.1, \sigma_3 = 0.125$, (b) $\sigma_1 = 0.05, \sigma_2 = 0.1, \sigma_3 = 0.2$, (c) $\sigma_1 = 0.1, \sigma_2 = 0.2, \sigma_3 = 0.4$, (d) $\sigma_1 = 0.1, \sigma_2 = 0.3, \sigma_3 = 0.9$.

S.1.4 Simulation study results: unbalanced noise across tissues and residual correlation between tissues

Here we show a more complicated simulation case in which the noise is unbalanced across tissues and the residual correlation varies between tissues. We use the same pattern of associations between responses and predictors as before with the mean level of the signal strength $\mu = 0.29$. For approximately half of the responses where the association is simulated, the residual correlation between tissues is high, with moderately imbalance of the signal-to-noise ratio (ratio 2.2 between strongest and weakest tissues), and the other half of responses have low correlation, but extremely high signal-to-noise ratio imbalance (ratio 15 between strongest and weakest tissues). The two residual correlation matrices are

$$\begin{pmatrix} 1.000 & 0.371 & 0.196 \\ 0.371 & 1.000 & 0.073 \\ 0.196 & 0.073 & 1.000 \end{pmatrix}, \begin{pmatrix} 1.000 & 0.027 & 0.027 \\ 0.027 & 1.000 & 0.001 \\ 0.027 & 0.001 & 1.000 \end{pmatrix}.$$

Figure S.3 shows in color scale the $p \times q$ matrices of regression coefficients B_ℓ for this simulation set-up. To give a further intuitive idea of the structure and amount of noise present in the simulations, we show in Figure S.4 values of the coefficient of determination R^2 for each response k and each tissue ℓ. In particular, we can see that signal is much stronger in tissue 1 than in tissues 2 and 3, and pinpoint which responses have a much weaker signal.

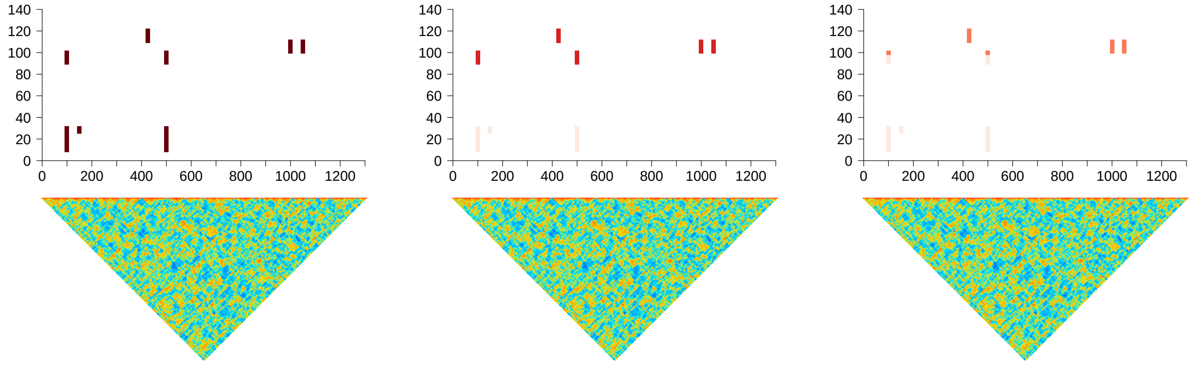

(a) Tissue 1 (b) Tissue 2 (c) Tissue 3

Figure S.3: Top: pictorial representation of the matrices of regression coefficients B_ℓ in tissues 1, 2 and 3. The entry (k, j) of B_ℓ indicates the strength of the association between SNP j and response k in tissue ℓ. Bottom: inverted triangles show one half of the correlation matrix of the SNP data.

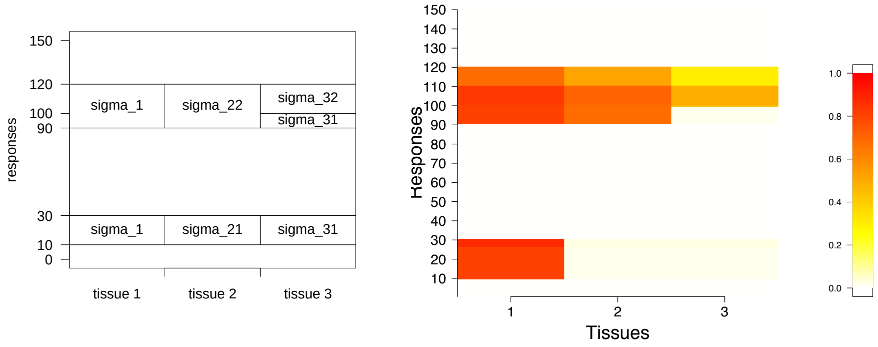

(a) (b)

Figure S.4: True signal structure in the simulation setting. (a) Diagram showing levels of tissue-specific noise standard error across the responses and tissues. Tissue-specific noise standard error levels are only indicated for the responses containing associations with the predictors. All other responses have "shared" noise standard error σ_{shared}. (b) For each response in each tissue, the value of the coefficient of determination R^2 used to simulate the data is represented by the colour scale

Figure S.5 shows the ROC curves of MT-HESS, MANOVA, iST-HESS and tissue-specific ST-HESS for 9 replications of this simulation set-up. We see the effect of the combination of the complex pattern with high and low values of residual correlation between tissues and extremely unbalanced signal-to-noise ratios across tissues: also in this complex situation MT-HESS outperforms MANOVA and iST-HESS.

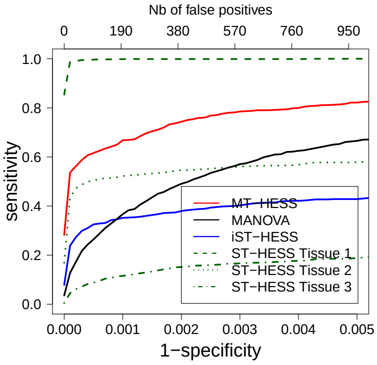

Figure S.5: ROC curves of MT-HESS, MANOVA, iST-HESS and tissue-specific ST-HESS with unbalanced signal-to-noise ratio across tissues and a complex pattern of residual correlation between tissues. Level of the signal strength $\mu = 0.29$.

Here we provide here detailed results for one replicate. Figure S.6 shows the heatmap of the matrices of associations. For MT-HESS (ST-HESS) the measure of association is the marginal posterior probability of inclusion that response k is associated to SNP j, whereas for the frequentist methods the measure is given by the p-value. The major observation is that MT-HESS (ST-HESS) provides a much better separation between noise and signal, whereas p-values corresponding to "one-at-a-time" frequentist methods exhibit a much more noisy pattern. In particular MANOVA (t-test) only detects the strongest signal in the third hotspot, while MT-HESS (ST-HESS) detects 5 out of 6 hotspots (see Figure S.7).

Regarding the information synthesis across multiple tissue, we see that MT-HESS (Figure S.6) tends to be conservative as it seeks agreement between the 3 tissues. This is why some associations reported in tissue 1 by ST-HESS are not present in the multiple tissue results. On the other hand, a few isolated false positives present in tissue 1 are not reported in the multiple tissue analysis.

On tissue 1, t-tests declare only one hotspot, whereas ST-HESS identifies 5 out of 6 hotspots. Also, ST-HESS manages to correctly find the relevant responses, and exclude the other ones (although there a few false positives for the fourth hotspot). On tissue 2, ST-HESS still manages to identify 5 out of 6 hotspots, although it detects fewer associations on hotspots 1 and 4. On tissue 3, both t-tests and ST-HESS fail to actually detect any hotspot, the reason being that this tissue has a very weak signal. Looking at the multiple-tissue analyses, we see that MANOVA reports the same hotspots and associations as tissue 1, that is MANOVA is fundamentally using only the information in tissue 1 to declare associations. On the other hand, MT-HESS combines the information of all tissues and dampens some of the associations found in tissue 1, mostly on hotspots 1 and 4. On the other hand, a few additional true associations are declared in hotspots 3 and 6.

This illustrates that MT-HESS is fairly conservative; in other words, borrowing information across tissues leads to reliable association detection, at the cost of possibly excluding signal arising solely in specific tissues. However when compared to multivariate MANOVA it is able to better separate signal from noise with better sensitivity and specificity.

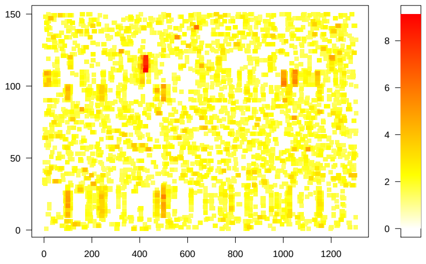

(a) t-test - Tissue 1

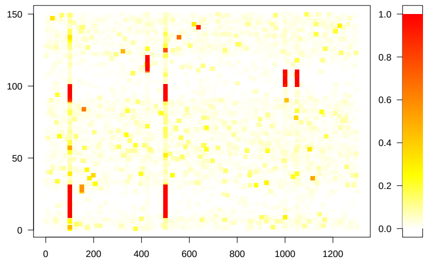

(b) ST-HESS - Tissue 1

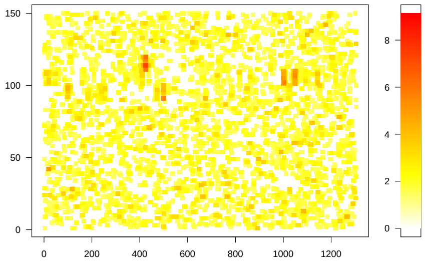

(c) t-test - Tissue 2

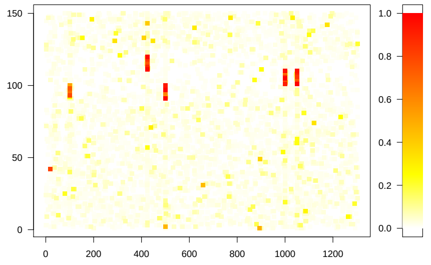

(d) ST-HESS - Tissue 2

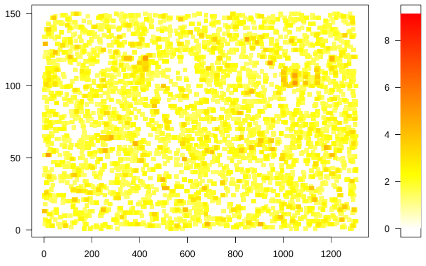

(e) t-test - Tissue 3

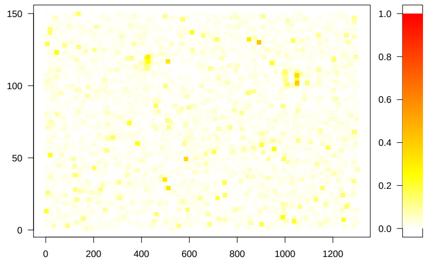

(f) ST-HESS - Tissue 3

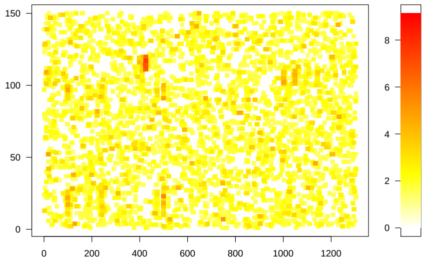

(g) MANOVA

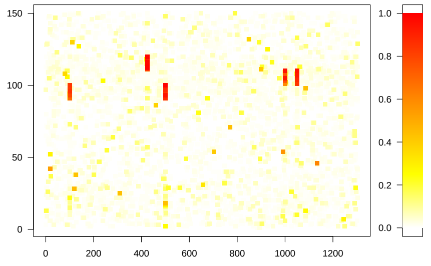

(h) MT-HESS

Figure S.6: Heatmaps of measures of associations for one replicate of the simulation study with unbalanced signal-to-noise ratio across tissues and a complex pattern of residual correlation between tissues. Level of the signal strength $\mu = 0.29$. Figures on the left and right show $-\log_{10}(p)$-values and marginal posterior probabilities of inclusion, respectively. The side bars indicate the colour key.

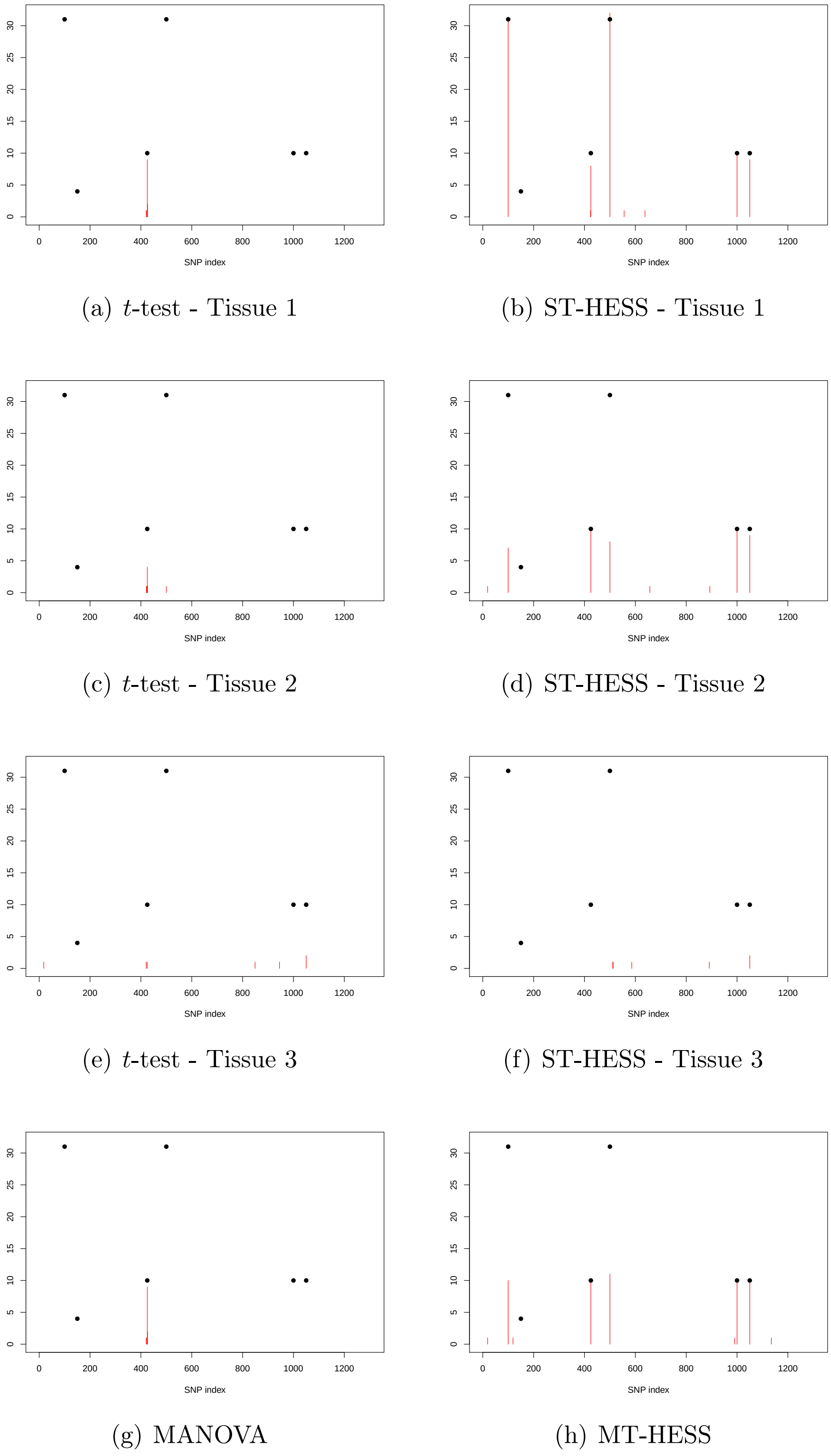

(a) t-test - Tissue 1

(b) ST-HESS - Tissue 1

(c) t-test - Tissue 2

(d) ST-HESS - Tissue 2

(e) t-test - Tissue 3

(f) ST-HESS - Tissue 3

(g) MANOVA

(h) MT-HESS

Figure S.7: Number of associations detected for one replicate of the simulation study with unbalanced signal-to-noise ratio across tissues and a complex pattern of residual correlation between tissues. Level of the signal strength $\mu = 0.29$. For each SNP (x-axis), the number of associations declared at a 5% FDR threshold is shown on the y-axis (red lines). Black dots represent the number of simulated associations for each SNP.

S.1.5 Simulation study results: strength of association signal

Figure S.8 shows ROC curves for simulated data with independent residuals between tissues, $\sigma_{\text{shared}} = 0$, and equal total noise standard deviation $\sigma_\ell^{\text{total}} = 0.1$, $1 \leq \ell \leq 3$. These are the same set-up as in Figure 2 (a) of the main text and Figure S.1 in the Supplementary Material, but here we use two levels of the signal strength $\mu = 0.29$, "strong association signal'̊), and $\mu = 0.09$ "weak association signal".

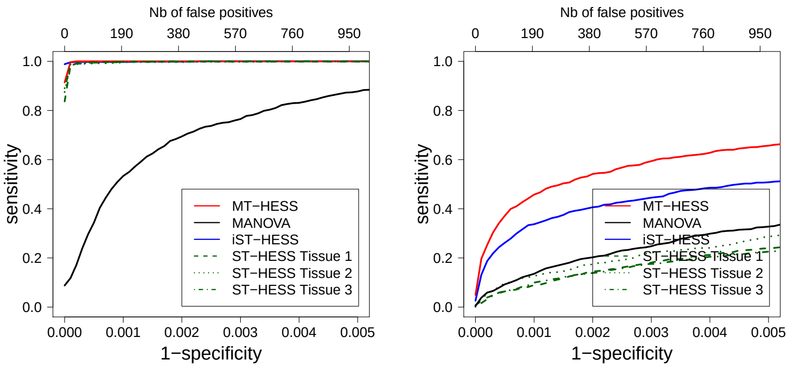

(a) (b)

Figure S.8: ROC curves of MT-HESS, MANOVA, iST-HESS and tissue specific ST-HESS for simulations with independent residuals between tissues ($\sigma_{\text{shared}} = 0$), balanced total noise standard deviation across tissues, $\sigma_\ell^{\text{total}} = 0.1$, $1 \leq \ell \leq 3$ and two levels of the signal strength. (a) "strong association signal" $\mu = 0.29$, (b) "strong association signal" $\mu = 0.09$.

S.1.5.1 Detailed results for "strong association signal"

This set-up is similar to the case shown in the main paper in Figure 2 (a), where noise is balanced across tissues $\sigma_\ell^{\text{total}} = 0.1$, $1 \leq \ell \leq 3$ and $\sigma_{\text{shared}} = 0$. We selected an arbitrary replicate for the case $\mu = 0.29$. Figure S.9 visualises the matrices of associations. As describe in Section S.1.4 both MT-HESS (ST-HESS) provides a much better separation between noise and signal. Additionally, despite the fact that $\mu = 0.29$ corresponds to a case with strong signal, MANOVA (and t-test) only seem to identify a cluster of association around SNP 400 and response 120, whereas MT-HESS (and ST-HESS) detect all the simulated clusters of associations. This analysis is confirmed when we look at the hotspots declaration on Figure S.10.

As described in the main text, to declare a hotspot, we first declare the "significant associations" by setting a threshold on the association matrices shown in Figure S.9. Because it is simulated data, we can calculate the exact FDR for each replicate, and we can set the threshold for each method (on MPPIs or p-values) to reach the same FDR rate of 5%. Once the significance threshold is set, for each SNP we count the number of significantly associated responses.

Figure S.10 shows a striking difference between the "one-at-a-time" approach and MT-HESS and ST-HESS, and confirms what was observed on the heatmaps. MANOVA and t-tests only identify one hotspot out of 6 (around SNP 400), while both ST-HESS and MT-HESS identify all the hotspots, with almost all the correct associations.

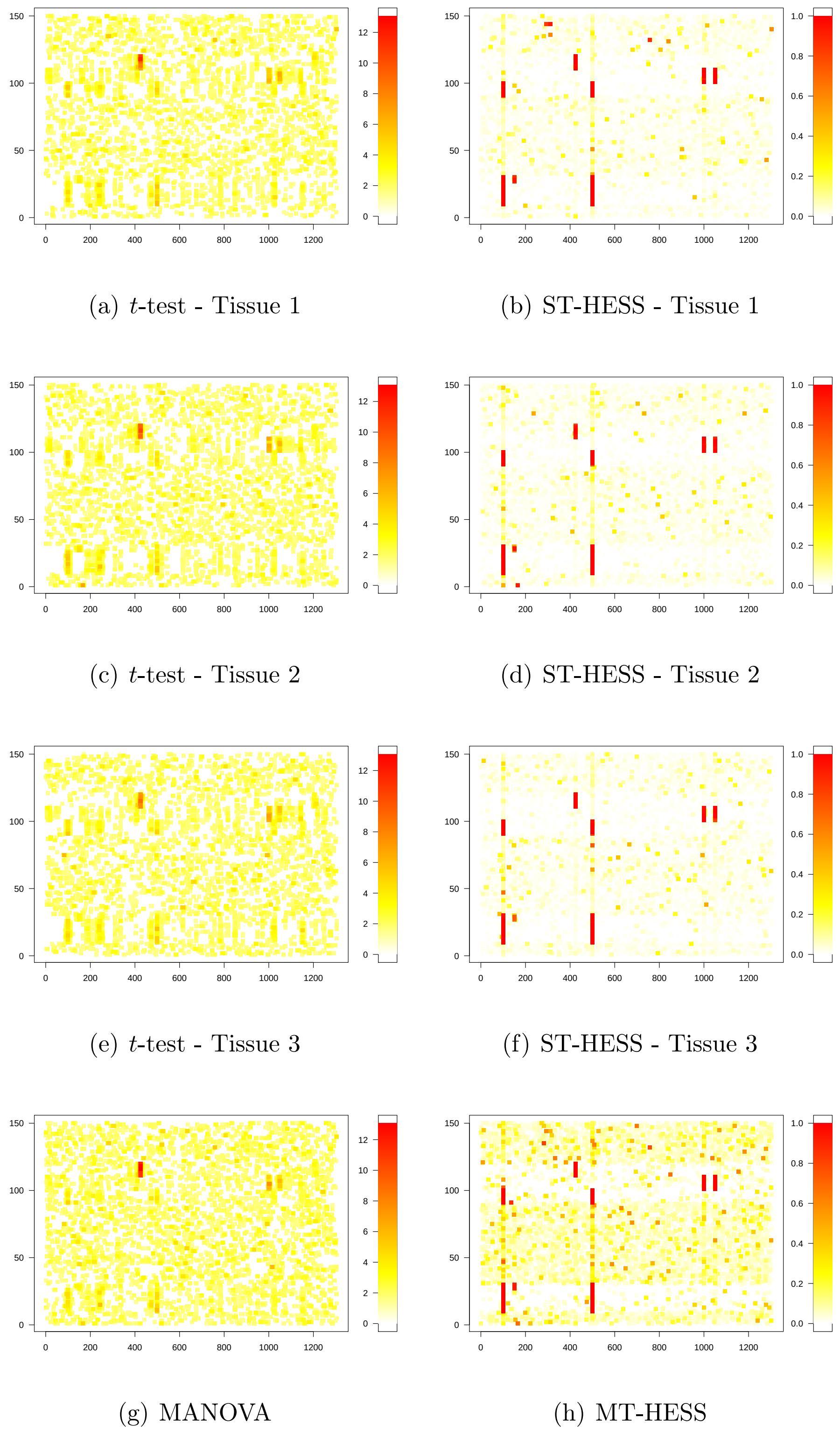

Figure S.9: Heatmaps of measures of associations for one replicate of the simulation study with balanced signal-to-noise ratio across tissues and no residual correlation between tissues. Level of the signal strength $\mu = 0.29$. Figures on the left and right show $-\log_{10}(p)$-values and marginal posterior probabilities of inclusion, respectively. The side bars indicate the colour key.

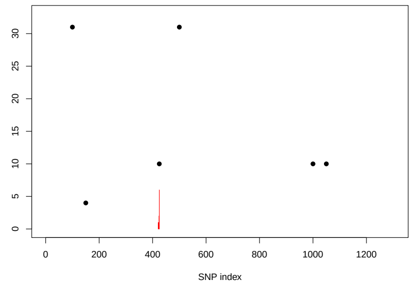

(a) t-test - Tissue 1

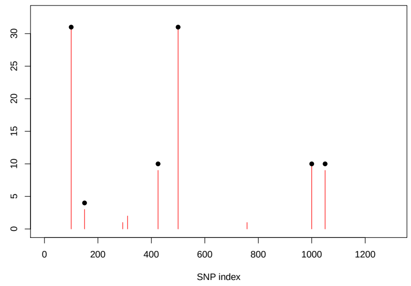

(b) ST-HESS - Tissue 1

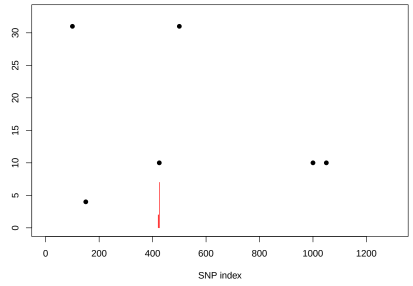

(c) t-test - Tissue 2

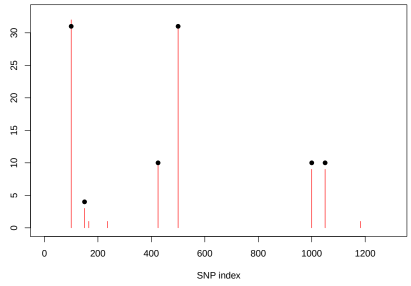

(d) ST-HESS - Tissue 2

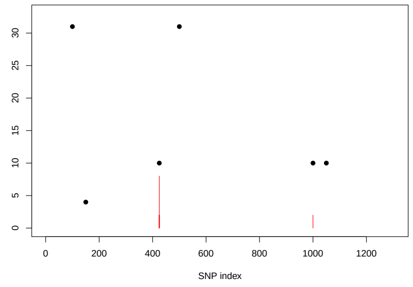

(e) t-test - Tissue 3

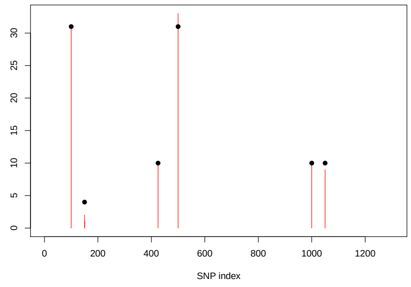

(f) ST-HESS - Tissue 3

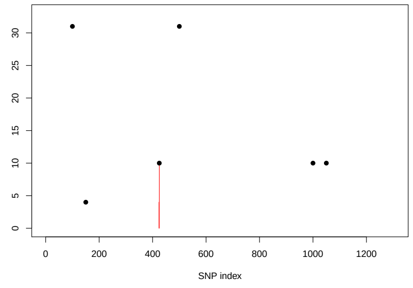

(g) MANOVA

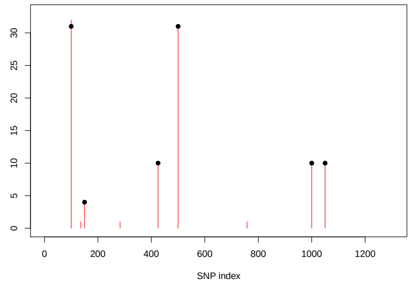

(h) MT-HESS

Figure S.10: Number of associations detected for one replicate of the simulation study with balanced signal-to-noise ratio across tissues and no residual correlation between tissues. Level of the signal strength $\mu = 0.29$. For each SNP (x-axis), the number of associations declared at a 5% FDR threshold is shown on the y-axis (red lines). Black dots represent the number of simulated associations for each SNP.

S.1.5.2 Detailed results for "weak association signal"

We display in this section the heatmaps of associations and the hotspots detection in the case of a weaker signal $\mu = 0.09$. Figures S.11 and S.12 show that no signal is detected by ST-HESS on any of the tissues, and similarly for the t-test. In contrast, MT-HESS manages to detect some associations corresponding to the two strongest hotspots (around SNPs 100 and 500).

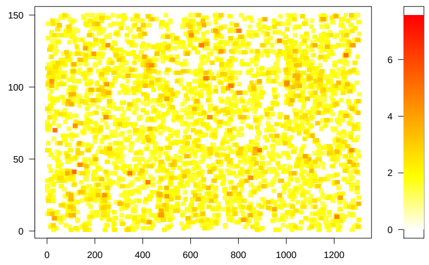

(a) t-test - Tissue 1

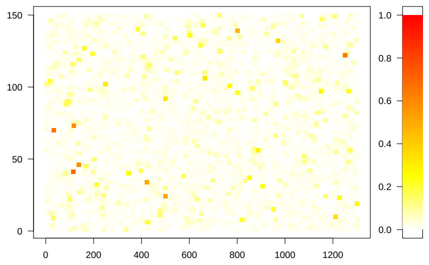

(b) ST-HESS - Tissue 1

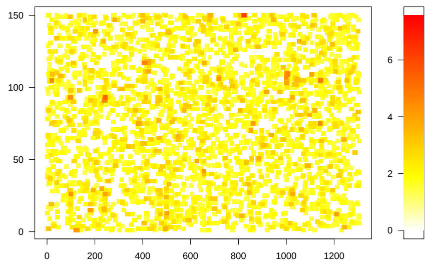

(c) t-test - Tissue 2

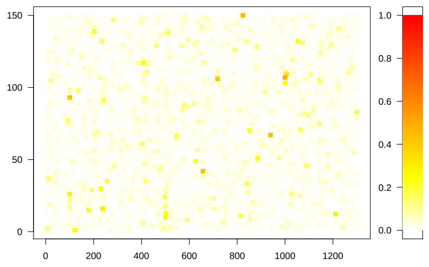

(d) ST-HESS - Tissue 2

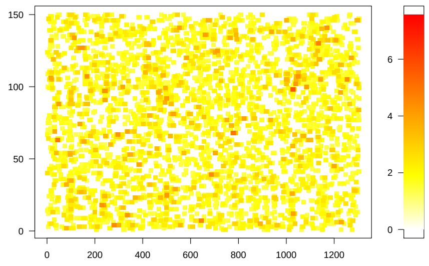

(e) t-test - Tissue 3

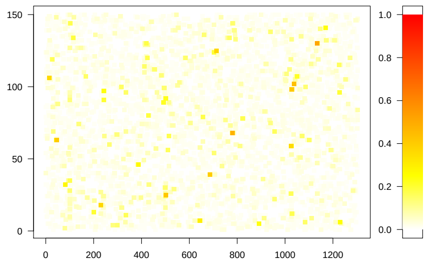

(f) ST-HESS - Tissue 3

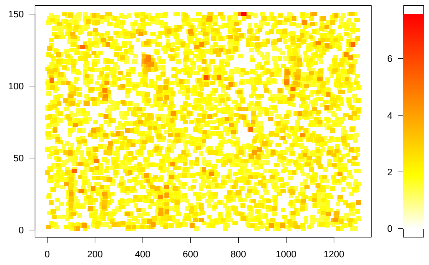

(g) MANOVA

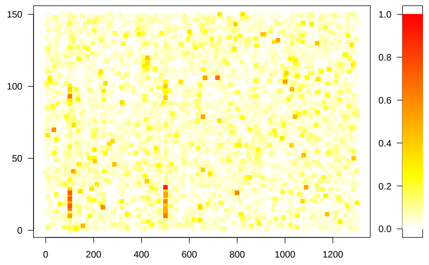

(h) MT-HESS

Figure S.11: Heatmaps of measures of associations for one replicate of the simulation study with balanced signal-to-noise ratio across tissues and no residual correlation between tissues. Level of the signal strength $\mu = 0.09$. Figures on the left and right show $-\log_{10}(p)$-values and marginal posterior probabilities of inclusion, respectively. The side bars indicate the colour key.

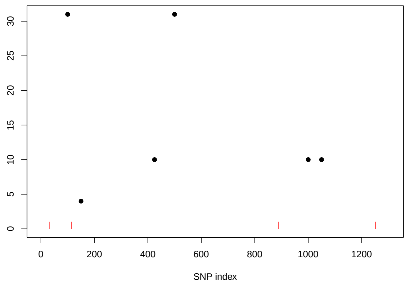

(a) t-test - Tissue 1

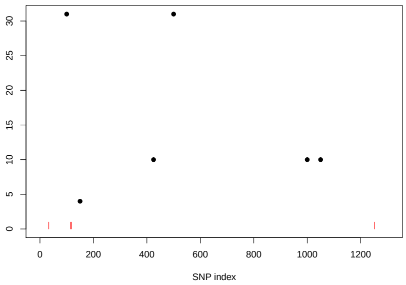

(b) ST-HESS - Tissue 1

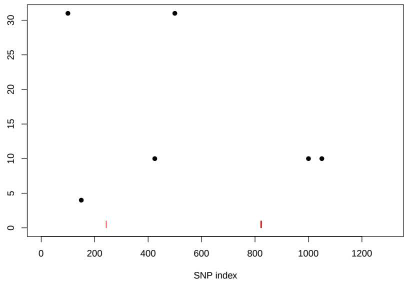

(c) t-test - Tissue 2

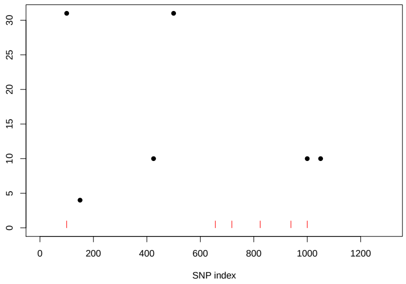

(d) ST-HESS - Tissue 2

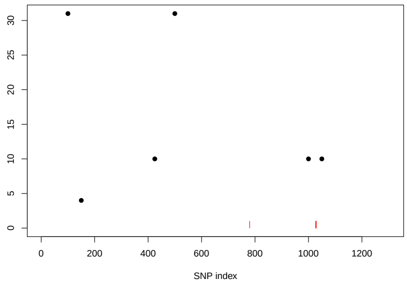

(e) t-test - Tissue 3

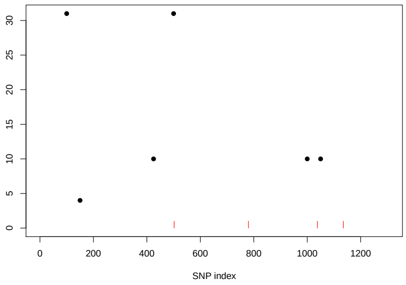

(f) ST-HESS - Tissue 3

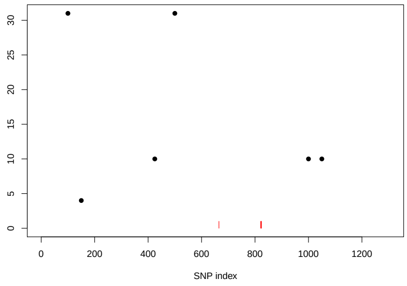

(g) MANOVA on all tissues

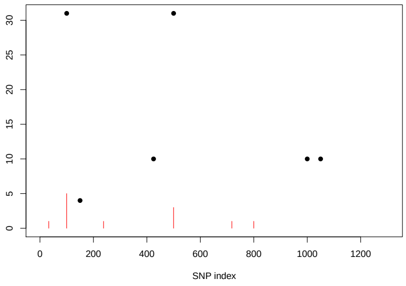

(h) MT-HESS

Figure S.12: Number of associations detected for one replicate of the simulation study with balanced signal-to-noise ratio across tissues and no residual correlation between tissues. Level of the signal strength $\mu = 0.09$. For each SNP (x-axis), the number of associations declared at a 5% FDR threshold is shown on the y-axis (red lines). Black dots represent the number of simulated associations for each SNP.

S.2 Post-processing for model probabilities

When calculating the most probable models for each response k, rather than using the empirical frequencies directly, we use the marginal likelihoods for the models visited during the MCMC chain, obtaining an estimate of the quantity

$$p(\gamma_k = \gamma | Y) = \frac{p(Y|\gamma_k = \gamma)p(\gamma_k = \gamma)}{\sum_{\gamma'} p(Y|\gamma_k = \gamma')p(\gamma_k = \gamma')}.$$

We use a Rao-Blackwellised estimator:

$$\begin{aligned} p(\gamma_k = \gamma|Y) &\approx \frac{1}{T}\sum_t p(\gamma_k = \gamma|Y, \theta^{(t)}) \\ &= \frac{1}{T}\sum_t \frac{p(Y|\gamma_k = \gamma, \theta^{(t)})p(\gamma_k = \gamma, \theta^{(t)})}{\sum_{\gamma'} p(Y|\gamma_k = \gamma', \theta^{(t)})p(\gamma_k = \gamma', \theta^{(t)})}, \end{aligned}$$

where $t = 1, \cdots, T$ are the MCMC iterations and $\theta^{(t)}$ stands for all parameters in the model other than γ_k.

The denominator is approximated by replacing the space of all possible model vectors γ' by the set of unique models visited during the MCMC chain $\{\gamma_{[1]}, \gamma_{[2]}, \cdots\}$, assuming that models not visited have low probability. Thus our estimator is

$$\widehat{p}(\gamma_k = \gamma_{[m]}|Y) = \frac{1}{T}\sum_t \frac{S_{kmt}}{\sum_{m'} S_{km't}},$$

where the index m ranges over the set of unique models visited. The quantity S_{kmt} can be simplified due to conditional independence in the model structure:

$$\begin{aligned} S_{kmt} &= p(Y_k|\gamma_k = \gamma_{[m]}, \theta^{(t)})p(\gamma_k = \gamma_{[m]}, \theta^{(t)}) \\ &= p(Y_k|\gamma_k = \gamma_{[m]}, g^{(t)})p(\gamma_k = \gamma_{[m]}, \{\omega_1, \cdots, \omega_q\}^{(t)}, \{\rho_1, \cdots, \rho_p\}^{(t)}). \end{aligned}$$

S.3 Calibration of FDR estimate

For our simulations we are able to calculate the true number of false positive and negative calls and thus check our estimates of the FDR. For each simulated data set, we find the marginal posterior probability of inclusion (MPPI) threshold corresponding to a given value of $bFDR$, and calculate the true FDR for that list of declared positives. Table S.1 shows the MPPI thresholds, the number of declared positives and the true FDR values for the simulations shown in Figure 2 in the main text. Values are averaged over multiple replications of each simulation case. Only replicates for which some positive associations are declared at that level are used. We give results for $bFDR$ of 5% and 10% as these are common values used for FDR thresholding.

The first two cases depicted in Fig. 2 (a) and (b) are high signal-to-noise ratio cases. Here we see that the $bFDR$ overestimates the true FDR, and so the decision rule is conservative. For Figure 2 (c) and (d), which have lower signal-to-noise ratio, the mean FDR is higher than the $bFDR$ estimate but the median is lower. For these cases where very few positives are declared, the number of possible true FDR values is very small, and it will be rare that the true FDR will match the $bFDR$ estimate. However the median values show that in these higher signal to noise cases we are still conservative.

	$bFDR = 5\%$				$bFDR = 10\%$			
	MPPI threshold	No. positives	True FDR (%) mean	True FDR (%) median	MPPI threshold	No. positives	True FDR (%) mean	True FDR (%) median
Fig. 2 (a)	0.87	48.0	0.64	0	0.70	73.1	4.8	4.2
Fig. 2 (b)	0.89	31.1	0.42	0	0.75	52.8	1.8	1.8
Fig. 2 (c)	0.90	1.4	0	0	0.84	2.9	15.6	0
Fig. 2 (d)	0.93	1.6	20	0	0.82	3.6	11.3	0

Table S.1: FDR calibration for the simulations used in the main text, for given values of the estimate $bFDR$. Numbers are averaged over all replications for which some positive associations are declared.

S.4 Sensitivity analysis

In this section we present a sensitivity analysis for the parameters c_{ρ_j} and d_{ρ_j} of the prior $\rho_j \sim \text{Gamma}(c_{\rho_j}, d_{\rho_j})$, $j = 1, \ldots, p$, that accounts for the propensity of predictor j to be a hotspot. Figure S.13 shows the shape of the gamma distribution for three different parameterization $c_{\rho_j} = d_{\rho_j} = 1.2$, $c_{\rho_j} = d_{\rho_j} = 2$ and $c_{\rho_j} = d_{\rho_j} = 5$. From the figure is clear that the larger the value of the parameters, the smaller the variance c_j/d_j^2 with the prior concentrating around its mean c_j/d_j. Note that with $c_{\rho_j} = d_{\rho_j} = 1.2$ we avoid placing substantial prior mass in 0 or shrinking large values of ρ_j towards 1.

Figure S.14 shows the results for different parameterizations of the prior $\rho_j \sim \text{Gamma}(c_{\rho_j}, d_{\rho_j})$ obtained in a replication of the simulation experiment illustrated in Figure 2 (a) (independent residuals, $\sigma_{\text{shared}} = 0$ and balanced case with $\sigma_\ell^{\text{total}} = 0.1$, $1 \leq \ell \leq 3$) and whose pattern of association is shown in Figure 1 (a). At bFDR ≤ 0.05, the parameterizations $c_{\rho_j} = d_{\rho_j} = 1.2$ and $c_{\rho_j} = d_{\rho_j} = 2$ produce nearly the same results, although we can already see that with $c_{\rho_j} = d_{\rho_j} = 2$ in the two hotspots simulated at chromosomes 14 and 15 the number of probe sets significantly associated starts to decrease. This is very clear with $c_{\rho_j} = d_{\rho_j} = 5$. Finally, note that with the parameterization $c_{\rho_j} = d_{\rho_j} = 1.2$, there are less false positives than in the other two cases as illustrated by the number of "small picks" close to the x-axis.

Figure S.15 shows sensitivity analysis for IDIN network example presented in Section 4.1. We confirm results obtained in the sensitivity analysis above with similar behavior with $c_{\rho_j} = d_{\rho_j} = 1.2$ and $c_{\rho_j} = d_{\rho_j} = 2$ parameterization. Both prior specifications detect three hotspots with the former sightly favoring more associations. Strikingly, with $c_{\rho_j} = d_{\rho_j} = 5$ almost no hotspots are detected showing the importance of our propensity prior and a parameterization that *a priori* allows large variance without placing the mode in 0.

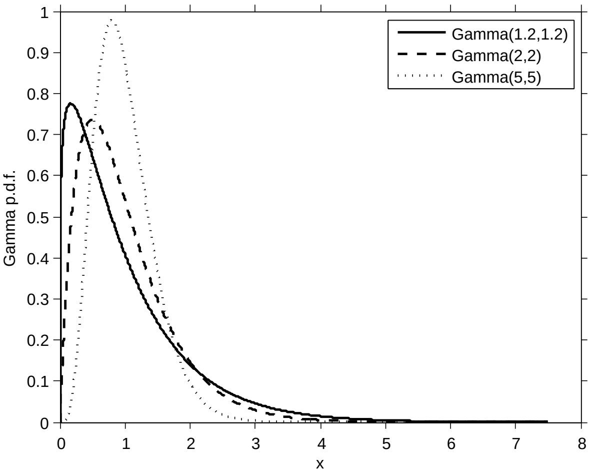

Figure S.13: Probability density function of the Gamma distribution with three different parameterizations, $c_{\rho_j} = d_{\rho_j} = 1.2$ (solid), $c_{\rho_j} = d_{\rho_j} = 2$ (dashed) and $c_{\rho_j} = d_{\rho_j} = 5$ (dotted)

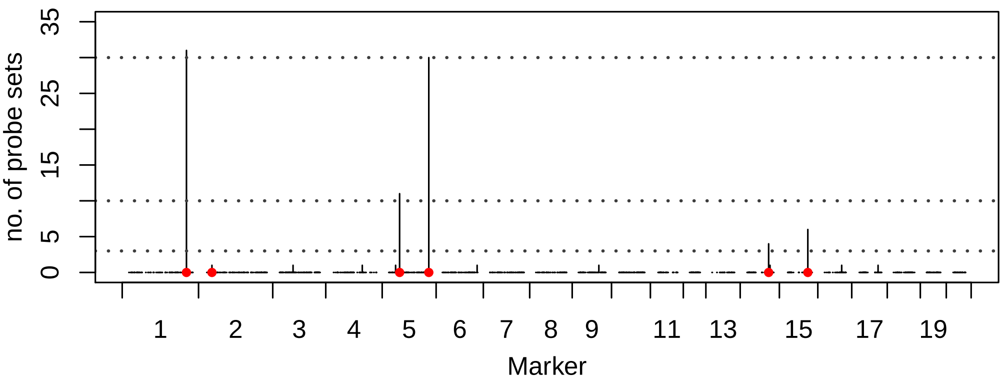

(a) $c_{\rho_j} = d_{\rho_j} = 1.2$

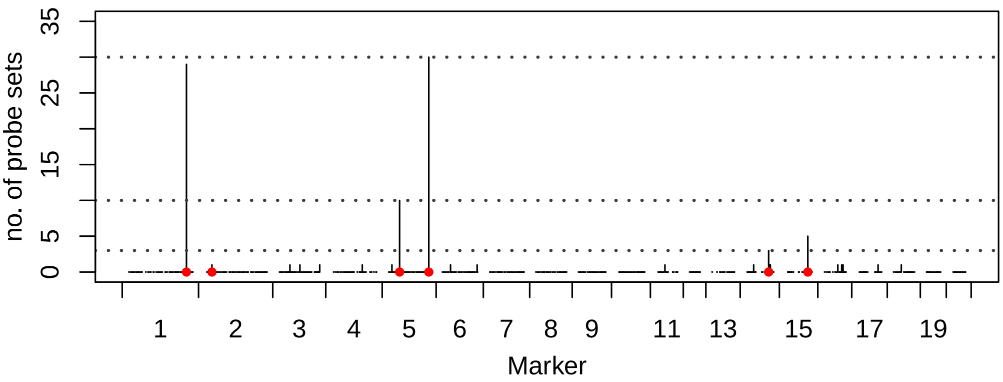

(b) $c_{\rho_j} = d_{\rho_j} = 2$

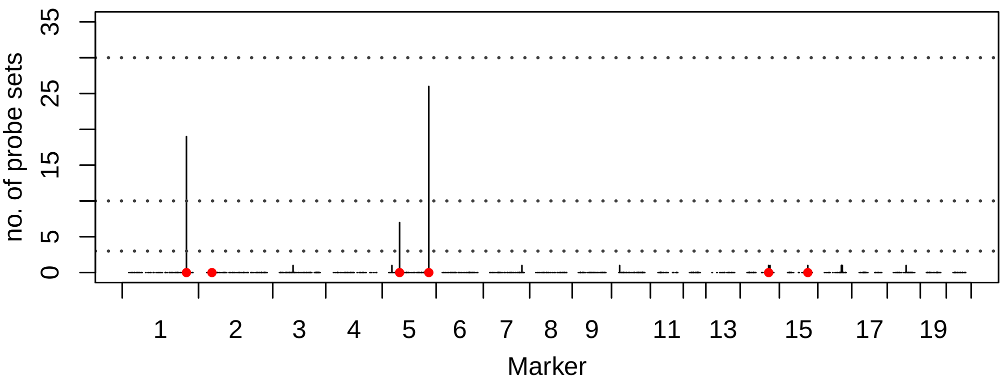

(c) $c_{\rho_j} = d_{\rho_j} = 5$

Figure S.14: Genome-wide Manhattan-like plots for one replication of the simulation experiment illustrated in Figure 2 (a) and whose pattern of association is shown in Figure 1 (a) for different parameterizations of the prior $\rho_j \sim \text{Gamma}(c_{\rho_j}, d_{\rho_j})$. The x-axis depicts chromosome number and position. The y-axis shows the number of probe sets significantly associated with each SNP (bFDR $\leq$ 0.05). Vertical dotted lines show the number of probe sets $(30, 3, 10, 30, 10, 10)$ associated with 6 hotspots marked with red dots in the x-axis.

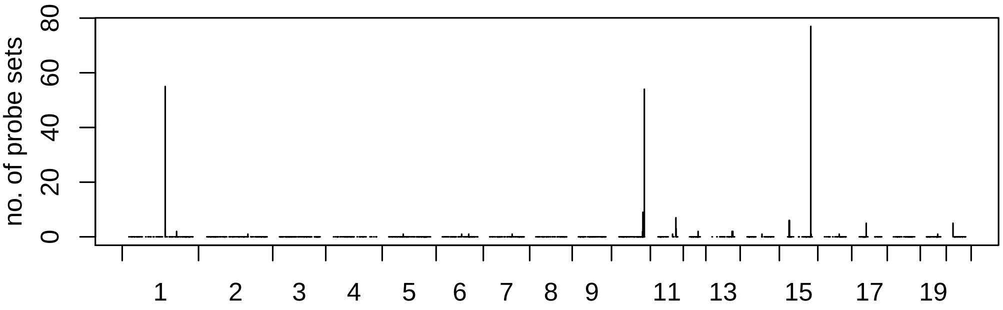

(a) $c_{\rho_j} = d_{\rho_j} = 1.2$

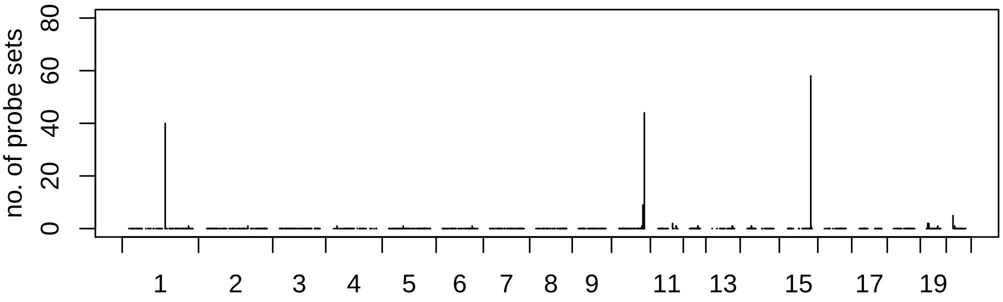

(b) $c_{\rho_j} = d_{\rho_j} = 2$

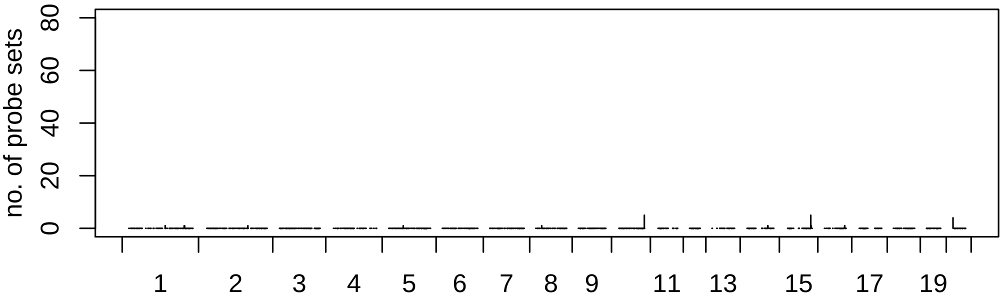

(c) $c_{\rho_j} = d_{\rho_j} = 5$

Figure S.15: Genome-wide Manhattan-like plots for the rat analysis showing the number of probe sets in the IDIN network associated with each SNP according to genomic location for different parameterizations of the prior $\rho_j \sim \text{Gamma}(c_{\rho_j}, d_{\rho_j})$. The x-axis depicts chromosome number and position. The y-axis shows the number of probe sets significantly associated with each SNP (bFDR ≤ 0.05).

S.5 Rat data results

Affymetrix RAE 230 2.0 microarrays were used for three tissues (left ventricle, aorta and liver). Gene expression summaries were derived using robust multichip average (RMA) algorithm (Irizarry *et al.*, 2003). To select informative probe sets targeting robustly expressed genes, for each of the three tissues we computed the mean expression over all normalized samples using the `normalmixEM` function from R package **mixtools** (Benaglia *et al.*, 2009). This function fits a mixture of two Gaussian distributions to the log-transformed mean expression (expressed and non/low- expressed genes). Starting values for the EM algorithm were chosen assuming 50% of expressed genes, and the initial mean values of the underlying Gaussian distributions were set equal to the first and third quartile of the gene expression distribution. The distribution with the lowest mean expression was considered as the non/low-expressed genes distribution, and a detection threshold was set, by taking the 95% percentile of the non/low-expressed genes distribution. Microarray probe sets above the detection threshold in less than two samples were considered as targeting non-expressed genes and filtered out from the analysis. Subsequently, an annotation-based filtering was performed to remove all probe sets that were non-specific or targeted non-Ensembl genes (Genome assembly: Rnor_5.0). The intersecting set of probe sets was obtained for the three tissues considered (left ventricle, aorta and liver), and the IDIN network (Heinig *et al.*, 2010) was retrieved (final number of probe sets = 146).

The intersecting probe sets of the IDIN network was mapped with HESS to the rat genome by using a non-redundant genome-wide SNP panel of 1,384 SNPs (Roider *et al.*, 2009). In this panel of rat SNPs, missing genotypes were imputed by using FastPhase (Scheet and Stephens, 2006). We carried out three single-tissue HESS runs (mapping the IDIN network to left ventricle, aorta and liver tissues separately), and a HESS multiple-tissue run by combining the IDIN network expression of these three tissues.

The PASTAA algorithm was used to identify overrepresented transcription factors binding sites motifs in the promoter of the corresponding human orthologs of the rat genes that were associated with the hotspot located in rat chromosome 10 with 5% Bayes FDR (Roider *et al.*, 2009). The rat genes associated to the regulatory hotspot located in rat chromosome 10 (52 protein coding genes) were mapped to human by considering only those genes that a one-one ortholog relationship in Ensembl 59 archive (which resulted in 41 human genes). PASTAA online tool was run with default parameterization and considering a promoter range of ±400 base pairs around the gene transcription start site. P-values were corrected for multiple testing by using the R package **p.adjust** and the adjustment method "Benjamini & Hochberg" (Benjamini, 1995). Corrected p-values are referred in the text as adjusted p-value. A transcription factor was considered to be significantly overrepresented if adjusted p-value ≤ 0.05. Bayes FDR was computing as explained in Section 3.4.

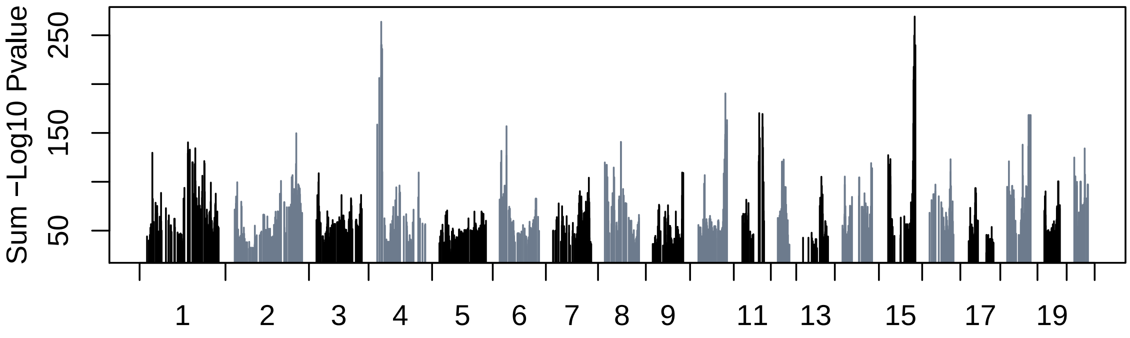

Figure S.16: Manhattan-like plot of the sum of the -$\log_{10}$ p-values from a MANOVA analysis of the rat data.

S.6 Human data results

S.6.1 Human data methods

S.6.1.1 Subjects

Adult patients with active inflammatory bowel disease (Crohn's disease or ulcerative colitis) were recruited from a specialist inflammatory bowel disease clinic at Addenbrooke's hospital in Cambridge, prior to commencing treatment as described previously (Lee *et al.*, 2011). Diagnosis was made using standard endoscopic, histopathological, and radiological criteria. Patients receiving immunomodulators or corticosteroids were excluded due to potential effects on gene expression. Ethical approval was for this work was obtained from the Cambridgeshire Regional Ethics Committee (REC08/H0306/21). All participants provided written informed consent.

S.6.1.2 Immune cell subsets

100 ml of whole blood was taken, and cell separations were performed as previously described (Lyons *et al.*, 2007).

S.6.1.3 Nucleic acid extraction

RNA was extracted from cell lysates using RNEasy Mini Kits (Qiagen) or Qiagen Allprep kits according to the manufacturer's instructions. RNA quality was assessed using an Agilent Bioanalyser 2100 (Agilent Technologies) and quantified by spectrophotometry using a NanoDrop ND-1000 spectrophotometer (Thermo Scientific). Genomic DNA was extracted using Qiagen Allprep kits according to the manufacturer's instructions and quantified as above.

S.6.1.4 Genomic DNA genotyping and genotype data QC

Genotyping was performed using the Illumina Human OmniExpress12v1.0 BeadChip at the Wellcome Trust Sanger Institute according to the manufacturer's protocol. After genotype calling with Illumina GenCall software, SNP data was processed in the following sequence using PLINK (Purcell *et al.*, 2007). Samples with a sex mismatch, abnormal heterozygosity, proportion of missing SNPs >0.05, and duplicated or related samples (identified using Identity by State) were removed. SNPs with missing calls >5%, extreme deviation from Hardy-Weinberg Equilibrium (p-value $<1\text{x}10^{-8}$), MAF <0.05 or which were monomorphic were removed. Finally, principal components analysis of the post-QC genotype calls combined with calls from HapMap3 founder individuals, using the R/Bioconductor **snpStats** package, confirmed all samples are of European ancestry.

Highly correlated SNPs were removed using the `--indep-pairwise` option in PLINK so that no pair of SNPs in a 50 SNP window shifted at 5 SNP intervals had r^2 >0.8.

S.6.1.5 Gene expression measurement, pre-processing and QC

200 ng RNA was processed for hybridization onto Affymetrix Human Gene ST 1.1 microarrays, according to the manufacturers instructions, prior to scanning. Microarrays were processed with the automated GeneTitan instrument which uses a 96 well plate. Chip probe intensity files were read into R version 3.02 (R Development Core Team, 2008) using the Bioconductor **oligo** package (Carvalho and Irizarry, 2010). Raw intensity values were pre-processed with the Robust Multi-array Average (RMA) algorithm which performs background correction, quantile normalization, $\log_2$ transformation and summarization to gene level via a median polish.

The **arrayQualityMetrics** package was run on the normalised expression matrices before and after batch correction with Combat (see below) and poor quality samples removed.

S.6.1.6 Correction for batch effects

Batch correction was performed using the `ComBat` function from the **sva** (Surrogate Variable Analysis) Bioconductor package (Leek *et al.*, 2012; Johnson *et al.*, 2007).

S.6.1.7 Filtering the expression data

Filtering of the expression data was performed prior to running HESS. Probesets on sex chromosomes and those which did not map to an Entrez gene ID were removed (Bioconductor **genefilter** package (Gentleman *et al.*, 2014)). The remaining probesets were then filtered by variance. We retained any probeset whose variance across samples was in the upper 10% in any one of the cell types.

S.6.1.8 Chromosome selection

We had no *a priori* knowledge of which chromosomes were most likely to contain hotspots. We selected chromosome 5 because a SNP (rs10044354) tagging an inflammatory bowel disease susceptibility locus has previously been shown to be a *cis* eQTL for *ERAP2* in both monocytes and B cells (Fairfax *et al.*, 2012).

S.6.2 Additional discussion

S.6.2.1 MT- and ST-HESS find the *cis* eQTL for *ERAP2* at an IBD susceptibility locus

We found one SNP, rs2549782, significantly associated with *ERAP2* expression, and this SNP was not associated with any other genes. The MPPI for the rs2549782-*ERAP2* association was 1 in the joint analysis and in all the separate cell type analyses. rs2549782 is in LD with rs10044354, the eSNP reported in (Fairfax *et al.*, 2012) ($r^2 = 0.72$ in 1000 Genomes CEU population). Unsurprisingly, HESS can find eQTLs associated with only one gene (typically *cis* effects which tend to be strong), as well as hotspots which are acting in *trans*. However, *cis* eQTLs can be detected by standard regression-based approaches, and the real advantage of HESS is the detection of trans-eQTL clusters (i.e. hotspots), and, in the case of MT-HESS, multi-tissue hotspots.

S.6.2.2 Hotspot associated with the second highest number of genes (using a 5% bFDR threshold)

The second "top" hotspot in CD4 T cells, rs17256961, was associated with 29 genes, and lies 35,469 base pairs from the top hotspot. These two SNPs are in LD with $r^2 = 0.58$ in 1000 Genomes CEU population. There was however no overlap in the genes associated with these SNPs. The visual representation of the MPPI matrix of SNP-gene associations around this locus in Supplementary Figure S.22 shows all MPPIs without any thresholding. The lack of noise is striking, and contrasts starkly with standard methods.

S.6.3 Additional figures

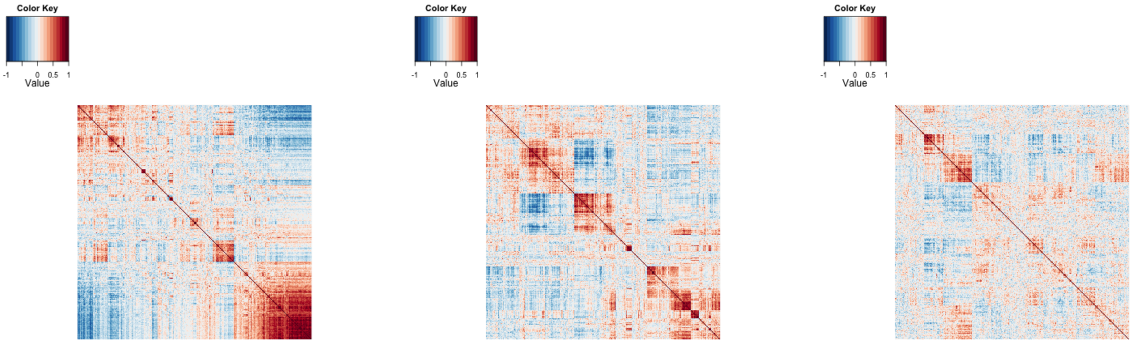

(a) CD4 T cells (b) CD8 T cells (c) monocytes

Figure S.17: Heatmap representations of gene-gene correlation matrices for the filtered expression data from each of the three cell types.

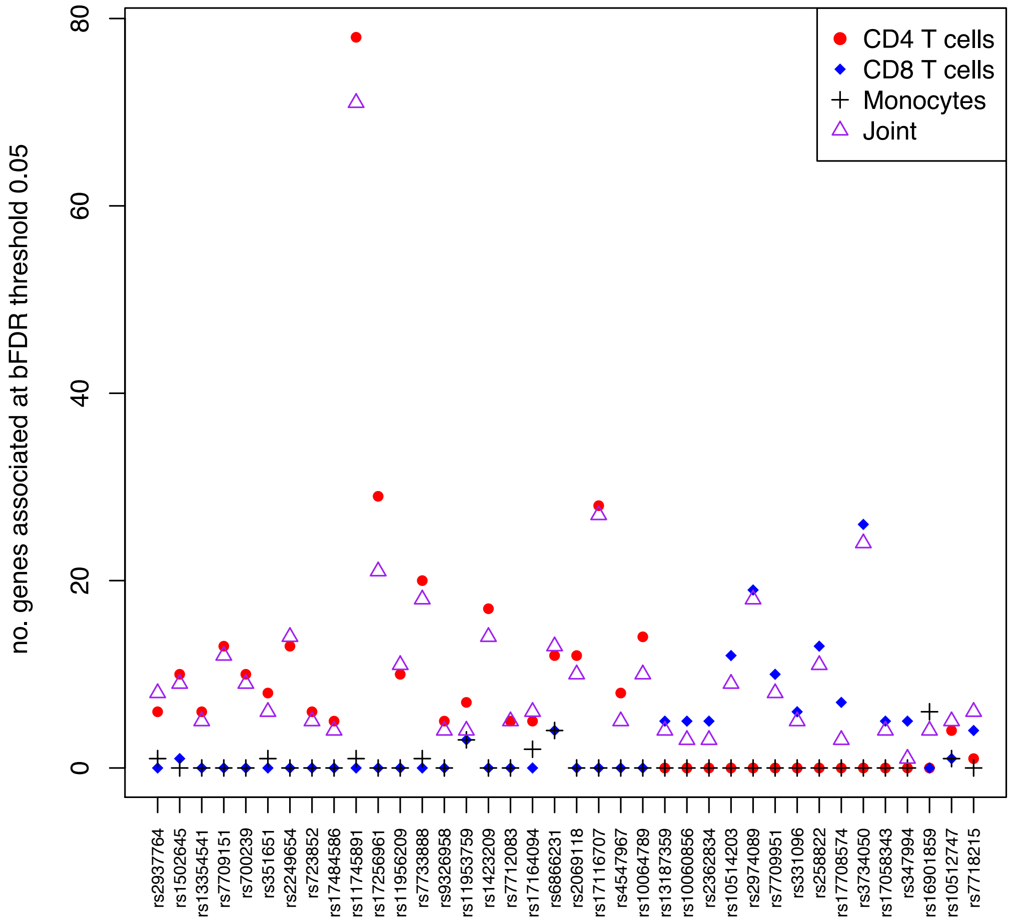

Figure S.18: SNPs with 5 or more associated genes (bFDR ≤ 0.05) in any one of the single tissues or in the multi-tissue analysis. We observe a few SNPs associated with more genes in the joint analysis compared to each of the single cell types analysed separately. For example, rs7718215 is associated with 6 genes in the joint analysis versus 1,4,0 for CD4 T cells, CD8 T cells and monocytes respectively in the single tissue analyses.

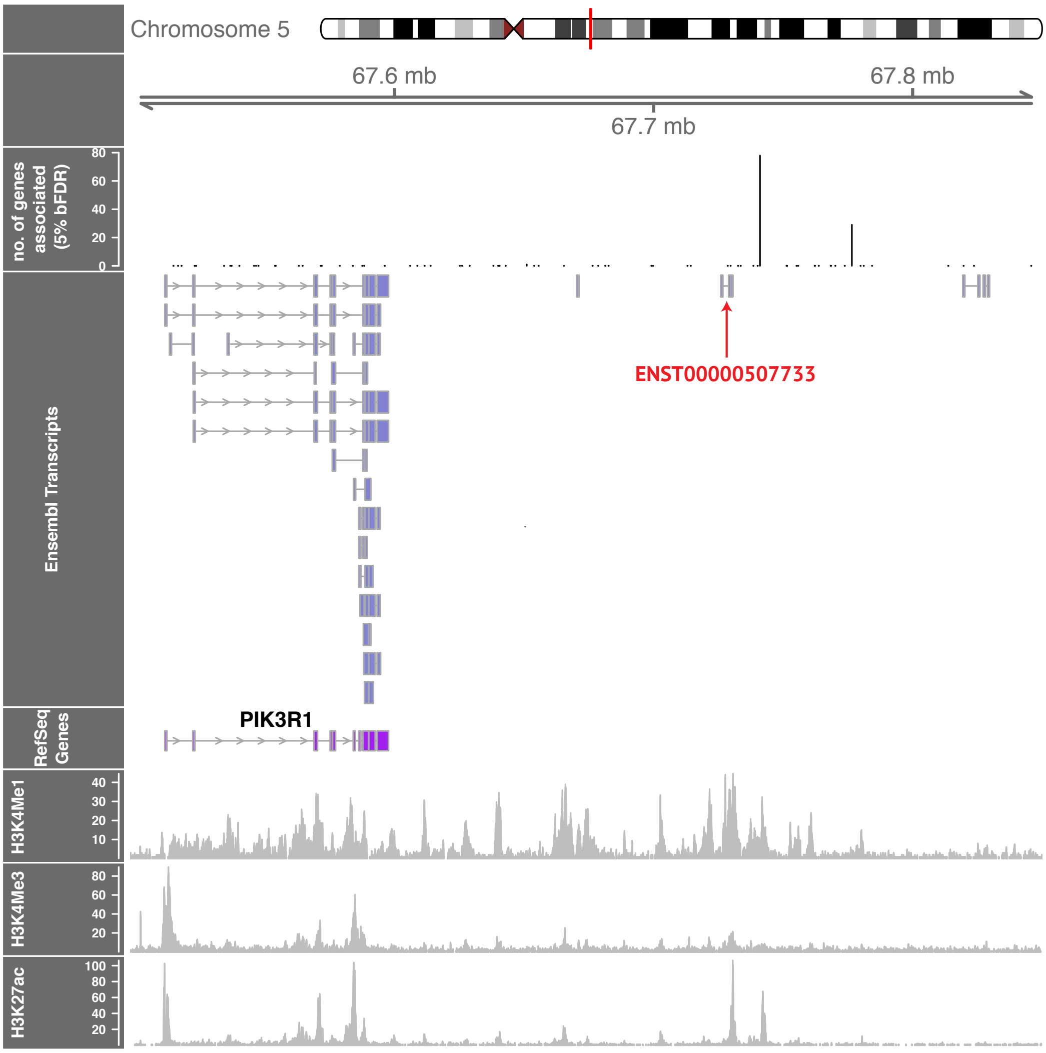

Figure S.19: Close up of genomic features in the region surrounding the hotspot SNP. Tracks from top to bottom: 1) Chromosome ideogram, with red line indicating location of display region. 2) Chromosomal position (hg19 genome build). 3) Manhattan plot showing the number of genes associated with each SNP (using a 5% bFDR threshold) in the displayed region (single tissue HESS- CD4 T cell analysis). The top two hotspots in CD4 T cells both lie in the region shown (black vertical lines). 4) Transcripts (including alternatively spliced isoforms) from Ensembl. The lincRNA *ENST00000507733*, indicated by the arrow and annotated in red, lies on the anti-sense strand. 5) RefSeq genes. 6-8) Histone marks from ENCODE. H3K4me1- histone monomethylation, which is associated with enhancer function, and with active transcription or with genes that are poised for activation. H3K4me3- histone trimethylation, which is linked to promoter activity. H3K27ac- histone acetylation, a marker of active enhancers.

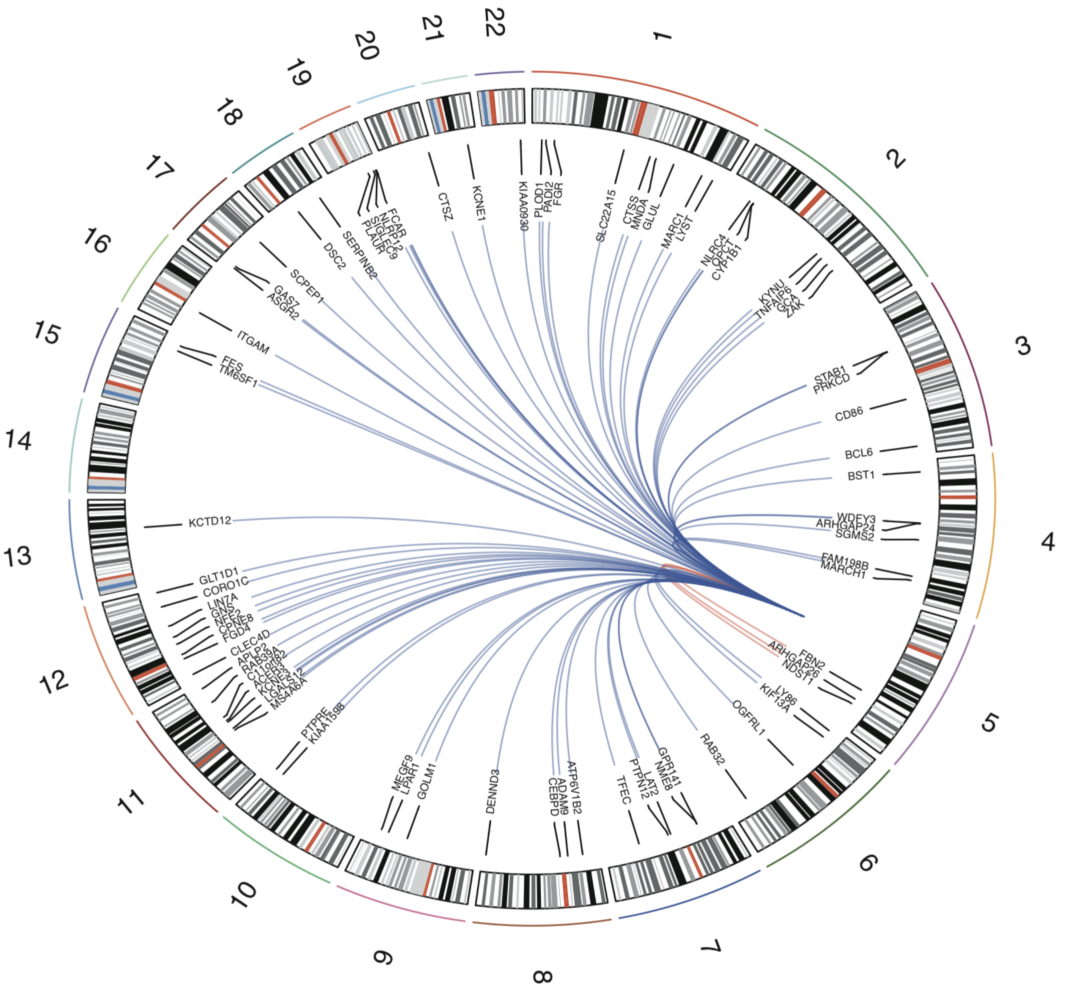

Figure S.20: Circos plot showing the hotspot SNP rs11745891 acting as a *trans* master regulator in CD4 T cells. Numbers (outermost) indicate chromosomes. Gene symbols (inner circle) indicate genes whose expression is significantly associated (Bayes FDR $\leq$0.05) with rs11745891. Interconnecting lines link the genomic location of the hotspot SNP and these genes (red lines for genes on the same chromosome as the SNP, blue lines for genes on different chromosomes).

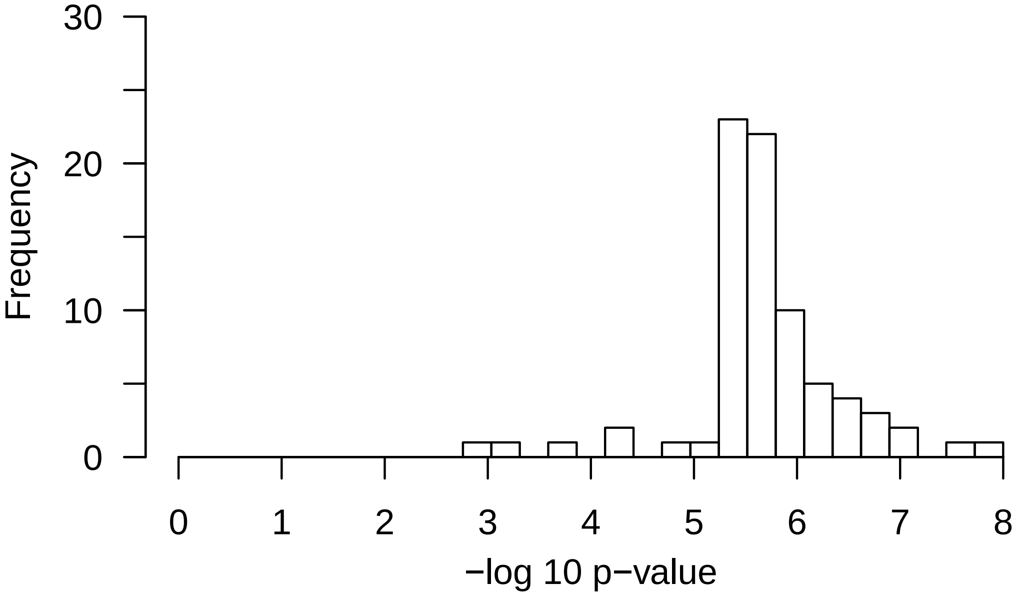

Figure S.21: Histogram of p-values from simple linear regression of expression of genes linked to hotspot SNP on rs11745891 genotype in CD4 T cells.

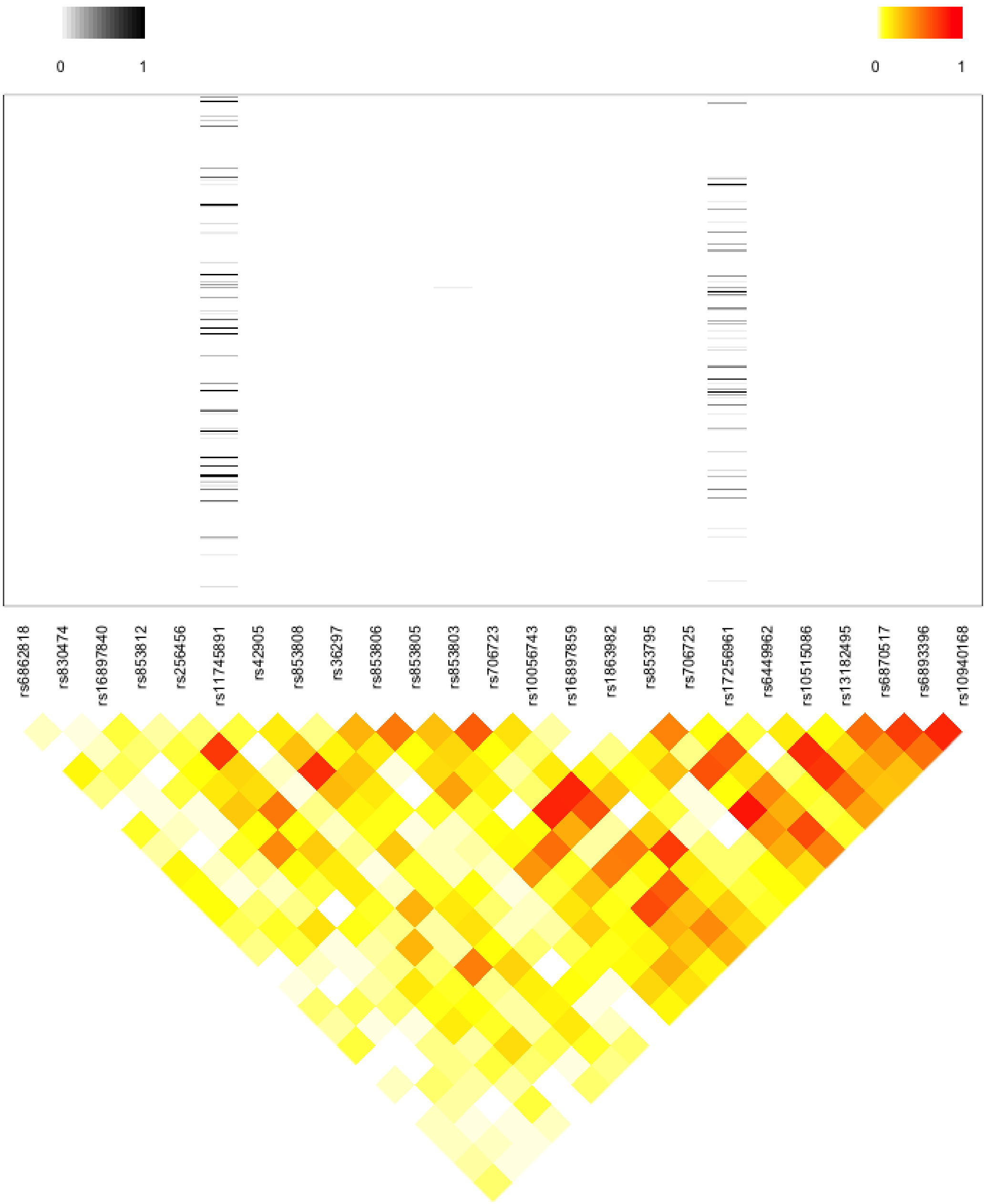

Figure S.22: Graphical representation of a subsection of the association signal (MPPI) matrix from ST-HESS in CD4 T cells (no thresholding). Rows are transcripts, columns are SNPs. SNPs appear in order of position on chromosome 5. The x-axis displays a zoomed in view of the columns of the MPPI matrix (representing SNPs) around the two hotspots, but all corresponding 3,248 rows (transcripts) are shown. Dark shading indicates strong signal (the MPPI shade key is shown in upper left hand corner). The inverted triangle shows LD structure in the region (r^2 indicated by the colour key in top right hand corner).

References

Benaglia, T., Chauveau, D., Hunter, D. R., and Young, D. (2009). mixtools: An R package for analyzing finite mixture models. *J. Stat. Softw.*, **32**(6), 1–29.

Benjamini, Y. (1995). Controlling the false discovery rate: a practical and powerful approach to multiple testing. *J. R. Stat. Soc. Ser. B*, **57**(1), 289–300.

Carvalho, B. S. and Irizarry, R. A. (2010). A framework for oligonucleotide microarray preprocessing. *Bioinformatics*.

Fairfax, B. P., Makino, S., Radhakrishnan, J., *et al.* (2012). Genetics of gene expression in primary immune cells identifies cell type-specific master regulators and roles of HLA alleles. *Nat. Genet.*, **44**(5), 502–510.

Gentleman, R., Carey, V., Huber, W., and Hahne, F. (2014). *Genefilter: methods for filtering genes from microarray experiments*. R package version 1.38.0.

Heinig, M., Petretto, E., Wallace, C., *et al.* (2010). A trans-acting locus regulates an anti-viral expression network and type 1 diabetes risk. *Nature*, **467**(7314), 460–4.

Irizarry, R. A., Hobbs, B., Collin, F., *et al.* (2003). Exploration, normalization, and summaries of high density oligonucleotide array probe level data. *Biostatistics*, **4**(2), 249–264.

Johnson, W. E., Li, C., and Rabinovic, A. (2007). Adjusting batch effects in microarray expression data using empirical Bayes methods. *Biostatistics*, **8**(1), 118–127.

Lee, J. C., Lyons, P. A., McKinney, E. F., Sowerby, J. M., *et al.* (2011). Gene expression profiling of CD8+ T cells predicts prognosis in patients with Crohn disease and ulcerative colitis. *J. Clin. Invest.*, **121**, 4170—79.

Leek, J. T., Johnson, W. E., Parker, H. S., Jaffe, A. E., and Storey, J. D. (2012). *sva: Surrogate Variable Analysis*. R package version 3.2.1.

Lyons, P. A., Koukoulaki, M., Hatton, A., *et al.* (2007). Microarray analysis of human leucocyte subsets: the advantages of positive selection and rapid purification. *BMC Genomics*, **8**, 64.

Purcell, S., Neale, B., Todd-Brown, K., *et al.* (2007). PLINK: a tool set for whole-genome association and population-based linkage analyses. *Am. J. Hum. Genet.*, **81**(3), 559–575.

R Development Core Team (2008). *R: A Language and Environment for Statistical Computing*. R Foundation for Statistical Computing, Vienna, Austria. ISBN 3-900051-07-0.

Roider, H. G., Manke, T., O'Keeffe, S., Vingron, M., and Haas, S. A. (2009). PASTAA: identifying transcription factors associated with sets of co-regulated genes. *Bioinformatics*, **25**(4), 435–42.

Scheet, P. and Stephens, M. (2006). A fast and flexible statistical model for large-scale population genotype data: applications to inferring missing genotypes and haplotypic phase. *Am. J. Hum. Gen.*, **78**(4), 629–44.
